# Copper Imbalance in Alzheimer’s Disease: Meta-Analysis of Serum, Plasma, and Brain Specimens, and Replication Study Evaluating *ATP7B* Gene Variants

**DOI:** 10.3390/biom11070960

**Published:** 2021-06-29

**Authors:** Rosanna Squitti, Mariacarla Ventriglia, Ilaria Simonelli, Cristian Bonvicini, Alfredo Costa, Giulia Perini, Giuliano Binetti, Luisa Benussi, Roberta Ghidoni, Giacomo Koch, Barbara Borroni, Alberto Albanese, Stefano L. Sensi, Mauro Rongioletti

**Affiliations:** 1 Molecular Markers Laboratory, IRCCS Istituto Centro San Giovanni di Dio Fatebenefratelli, 25125 Brescia, Italy; cbonvicini@fatebenefratelli.eu (C.B.); lbenussi@fatebenefratelli.eu (L.B.); rghidoni@fatebenefratelli.eu (R.G.); 2 Fatebenefratelli Foundation for Health Research and Education, AFaR Division, San Giovanni Calibita Fatebenefratelli Hospital, Isola Tiberina, 00186 Rome, Italy; mariacarla.ventriglia@afar.it (M.V.); ilaria.simonelli@afar.it (I.S.); 3 Unit of Behavioral Neurology, IRCCS Mondino Foundation, 27100 Pavia, Italy; alfredo.costa@unipv.it (A.C.); giulia.perini01@universitadipavia.it (G.P.); 4 Department of Brain and Behavior, University of Pavia, 27100 Pavia, Italy; 5 MAC Memory Clinic and Molecular Markers Laboratory, IRCCS Istituto Centro San Giovanni di Dio Fatebenefratelli, 25125 Brescia, Italy; gbinetti@fatebenefratelli.eu; 6 Section of Human Physiology, University of Ferrara, 44121 Ferrara, Italy; g.koch@hsantalucia.it; 7 Department of Clinical and Behavioural Neurology, Santa Lucia Foundation IRCCS, 00179 Rome, Italy; 8 Centre for Neurodegenerative Disorders, Department of Clinical and Experimental Sciences, University of Brescia, 25123 Brescia, Italy; barbara.borroni@unibs.it; 9 Department of Neurology, IRCCS, Istituto Clinico Humanitas, Rozzano, 20089 Milan, Italy; alberto.albanese@humanitas.it; 10 Department of Neuroscience, Imaging and Clinical Science, “G. D’Annunzio” University of Chieti-Pescara, 66100 Chieti, Italy; 11 Institute for Mind Impairments and Neurological Disorders—iMIND, University of California—Irvine, Irvine, CA 92697, USA; 12 Molecular Neurology Units, Center for Advanced Studies and Technology (CAST), University G. D’Annunzio of Chieti-Pescara, 66100 Chieti, Italy; 13 Department of Laboratory Medicine, Research and Development Division, San Giovanni Calibita Fatebenefratelli Hospital, Isola Tiberina, 00186 Rome, Italy; maurociroantonio.rongioletti@fbf-isola.it

**Keywords:** Alzheimer’s disease, Alzheimer’s dementia, Cu, ceruloplasmin, meta-analysis, brain, serum, ATP7B, Wilson’s disease

## Abstract

Evidence indicates that patients with Alzheimer’s dementia (AD) show signs of copper (Cu) dyshomeostasis. This study aimed at evaluating the potential of Cu dysregulation as an AD susceptibility factor. We performed a meta-analysis of 56 studies investigating Cu biomarkers in brain specimens (pooled total of 182 AD and 166 healthy controls, HC) and in serum/plasma (pooled total of 2929 AD and 3547 HC). We also completed a replication study of serum Cu biomarkers in 97 AD patients and 70 HC screened for rs732774 and rs1061472 *ATP7B*, the gene encoding for the Cu transporter ATPase7B. Our meta-analysis showed decreased Cu in AD brain specimens, increased Cu and nonbound ceruloplasmin (Non-Cp) Cu in serum/plasma samples, and unchanged ceruloplasmin. Serum/plasma Cu excess was associated with a three to fourfold increase in the risk of having AD. Our replication study confirmed meta-analysis results and showed that carriers of the *ATP7B* AG haplotype were significantly more frequent in the AD group. Overall, our study shows that AD patients fail to maintain a Cu metabolic balance and reveals the presence of a percentage of AD patients carrying *ATP7B* AG haplotype and presenting Non-Cp Cu excess, which suggest that a subset of AD subjects is prone to Cu imbalance. This AD subtype can be the target of precision medicine-based strategies tackling Cu dysregulation.

## 1. Introduction

Alzheimer’s dementia (AD) is a multifactorial condition for which a new disease-modifying therapy, aducanumab, has been recently approved by FDA, even though post-approval studies have been requested to demonstrate its clinical efficacy [1,2,3]. Hallmarks of AD are extracellular deposits of the beta-amyloid protein (Aβ), intraneuronal aggregates of the Tau protein, and reactive gliosis [4,5]. Many modifiable risk factors contribute to shaping the disease onset and progression [6]. In their analysis of primary prevention of AD, Norton et al. 2014 [6], extending studies from Barnes and Yaffe 2011 [7], excluded some potential modifiable risk factors from their estimation of a population-attributable risk (PAR) based on relative risks from existing meta-analyses and prevalence of these risk factors [1]. PAR for dietary factors and metal imbalance has been in fact impeded since data regarding population prevalence of abnormal values were missing in the literature. Unfortunately, this initial gap contributed to hampering the full exploration of the hypothesis that metals might be modifiable risk factors for AD. Almost a decade ago, a novel metal hypothesis [8] and several meta-analysis studies (reviewed in [9]) had triggered a new interest in the pathogenic interaction between metals, mainly iron (Fe), copper (Cu), and zinc (Zn), and a set of AD-related proteins (primarily belonging to the Amyloid beta-Precursor Protein (AβPP)/Aβ system). Cu is an essential micronutrient, the catalyst or component of many metalloproteins or enzymes that help to control cellular life and energy production in a variety of biological systems. Nonbound ceruloplasmin (Non-Cp) Cu (also known as ‘free copper’) is the fraction of Cu in serum/plasma that does not bind to ceruloplasmin, the main protein that carries Cu in the blood (reviewed in [9]). The expansion of the blood pool of Non-Cp Cu is toxic as, in this form, the metal can cross the blood–brain barrier (BBB), accumulate as “labile Cu” in the brain [10], and participate in a variety of harmful and cell-damaging activities [9], as exemplified by Wilson’s disease (WD), a rare autosomal recessive disorder caused by mutations of *ATP7B*, the gene encoding for ATPase7B, a Cu pump located in hepatocytes, and endothelial cells of the BBB. Normally, Cu acts beneficially as a catalyst and critical component of metalloproteins and enzymes essential for cellular and brain functioning, such as the systemically important antioxidant Cu/Zn-Superoxide dismutase (SOD1), and cytochrome C oxidase, which produces neuron energy in the mitochondria. However, when not properly bound, Cu undergoes redox cycling reactions with O_2_, resulting in the catalytic production of reactive oxygen species (ROS) of which H_2_O_2_ can diffuse through cell membrane and then produce the very reactive hydroxyl radical (HO•), catalyzed by Cu (Fenton-type reactions) [11]. In the past decade, many studies have uncovered a link between AD pathogenesis and Cu dysmetabolism (reviewed in [12]): AβPP/Aβ are Cu binding proteins with a potential role as natural Cu buffering proteins, and Cu^2+^ binding dramatically changes Aβ aggregation propensity, structure, and toxicity [13], with a plethora of effects spanning from reducing energy production in mitochondria to altering synaptic function and cognitive deterioration (reviewed in [12]), as revealed by preclinical models of chronic Cu exposure [14,15,16,17]. Furthermore, mutations in the genes involved in Aβ buildup and processing (AβPP, PSEN1/PSEN2) have been reported to disturb the metal-buffering AβPP/Aβ system [12]. The ATPase7B pump loads Cu onto nascent ceruloplasmin in hepatocytes and onto the glycophosphatidyl inositol ceruloplasmin in astrocytes (reviewed in [12]). Defects in these processes lead to increased release of Non-Cp Cu in the blood and activate cell-damaging events related to AD (reviewed in [12]).

Meta-analysis on Cu in brain specimens [18] and serum samples [19] as well as on serum Non-Cp Cu [20] and ceruloplasmin [21] have been published, but they appear a little dated. Furthermore, despite this vast literature, the scientific community has not yet reached consensus on the role played by Cu in AD. Indeed, the topic and the clinical significance of Cu in AD are still considered controversial, probably as many studies on brain specimens have reported decreased Cu values in AD, while those on circulating Cu reported increased values, allowing contrasting interpretations. To help clarify the evidence, we aimed at summarizing the available case-control studies produced on brain and on circulating Cu in AD in a comprehensive meta-analysis to determine the associations of a panel of Cu biomarkers with the disease. 

We also performed a replication study intended at evaluating levels of serum Cu, Non-Cp Cu, ceruloplasmin concentrations, ceruloplasmin activity, ceruloplasmin specific activity (iCp/eCp), and the Cu to ceruloplasmin ratio (Cu:Cp) in a sample of 97 AD patients and 70 healthy controls (HC). Individuals were screened for the two functional single nucleotide polymorphisms (SNPs) rs1061472 and rs732774 of *ATP7B* [11], associated with an increased risk for AD [22]. The role played by these SNPs was reported in a recent study [23]. The SNP amino-acid substitutions K832R and R952K modulate the ATPase7B properties in vitro and alter serum Cu status *in vivo*. rs1061472 and rs732774 *ATP7B* SNPs primarily affect the ATPase7B abundance and reduce its trafficking in response to elevated Cu [23].

## 2. Materials and Methods

### 2.1. Meta-Analysis

#### 2.1.1. Search Strategy

To identify appropriate studies for the meta-analysis, we followed the procedural steps indicated by Cochrane (http://www.cochrane-handbook.org; accessed date 1 July 2019). To this aim, we entered in PubMed the keywords “Alzheimer’s disease”, “Alzheimer’s dementia”, “Cu”, “serum”, “plasma”, “brain”, “metals”, and their combinations and selected studies published from January 1984 to July 2020. We also identified other studies by using the “Scopus” and “ISI Web of Knowledge” databases. In addition to the reviewing of contents of this first selection of studies, we ran through their reference lists to search for additional studies via Google Scholar. We considered only studies that took into account comparative analyses between AD and HC that were reporting original results in peer-reviewed journals. In most studies, the severity of the AD-related cognitive decline was assessed by the Mini-Mental State Examination (MMSE). The target of our meta-analysis was the comparison, between AD patients and HC, of Cu levels as reported in the selected dataset of papers. 

#### 2.1.2. Data Extraction and Manipulation for Meta-Analysis

Two review authors (MC and IS) independently extracted data from the included studies by collecting them in an excel sheet. Any divergence was resolved through discussion. 

Overall, we extracted the number of participants in each group of each selected study, characteristics of participants (age and sex), and type of study. Concerning the parameters of interest, we extracted means and standard deviations (SD) related to the AD and HC groups. When this information was not available, we tried to extract medians and intervals (range or interquartile range) and insert them into the tables.

When median and IQR or Min-Max were extracted, data manipulation was necessary to estimate mean SD. From these values, means and SD were calculated by methods published by Hozo et al. [24] or Wan et al. [25]. SD was also calculated in line with the indication of the Cochrane Handbook [26].

#### 2.1.3. Statistical Procedures Applied to Run Meta-Analysis

Four studies of meta-analysis were performed that took into consideration (i) levels of Cu in the brain, (ii) both Cu and (iii) Non-Cp Cu, and (iv) ceruloplasmin in serum/plasma of the two study groups (AD and HC). Meta-analysis was performed by applying a random-effects model to obtain a pool of standardized mean difference (SMD) by the method of Hedges. Both study-wise and group-wise analyses were performed. The data heterogeneity was evaluated via Cochran’s Q test and quantified through the I^2^. The I^2^ describes the rate of variation across studies due to heterogeneity rather than chance. The parameter ranges from 0 (indicating no heterogeneity) to 100 (equal to maximal heterogeneity). 

A funnel plot was used to investigate publication bias. The Egger’s test for funnel plot asymmetry was used only when there were at least 10 studies included in the meta-analysis [26]. 

In a meta-analysis of Cu in the brain, subgroup analysis was performed stratified for the hippocampus, amygdala, a not well defined ‘cerebral cortex’ area, and frontal cortex.

In a meta-analysis of Non-Cp Cu, a sensitivity analysis was performed, excluding studies that have a Cu:Cp ratio in HC lower than 6 and higher than 8.

In a meta-analysis of total copper and Non-Cp Cu, meta-regression analysis was performed to evaluate the effect on the pooled SMD of the difference in mean age between HC and AD patients. 

A *p*-value < 0.05 was considered statistically significant. All statistical analyses were performed with STATA v10.

### 2.2. Replication Study Investigating Differences in Serum Cu Biomarkers between AD and HC

#### 2.2.1. Subjects Analyzed in the Replication Study

Ninety-seven consecutive AD patients and 70 cognitively normal individuals were included in a replication study. Alzheimer’s disease patients [27,28] with a Mini-Mental State Examination (MMSE) score of 25 or less [29] were enrolled by two specialized neurological and dementia care centers in Italy—the Memory Clinic of the IRCCS Istituto Centro San Giovanni di Dio, Fatebenefratelli, Brescia, Italy, and Dipartimento di Scienze del Sistema Nervoso e del Comportamento, Università di Pavia, Istituto Neurologico Nazionale IRCCS C. Mondino—using common standardized clinical protocols and guidelines. Healthy controls were individuals with no sign of neurological disorders and with normal cognitive function, selected mainly among spouses. Exclusion criteria were conditions affecting Cu metabolism, evaluated on the basis of past medical history as reported in detail elsewhere [30]. Participants in the replication study underwent blood sampling while fasting, analyses of an extensive panel of Cu metabolism markers composed by serum Cu, non-ceruloplasmin Cu, ceruloplasmin concentration, ceruloplasmin activity, ceruloplasmin specific activity (iCp/eCp), ceruloplasmin to Cu ratio (Cu:Cp) ratio, DNA extraction, and genotyping of *ATP7B* rs1061472 and rs732774.

#### 2.2.2. Sample Collections

Fasting blood samples were collected in the morning and sera samples were separated by centrifugation (3000 rpm, 10 min, and 4 °C). They were then divided into 0.5 mL aliquots and rapidly stored at −80 °C. Biological samples, isolated according to standard procedures, were stored at Fatebenefratelli Biobank (IRCCS Centro San Giovanni di Dio Brescia, Italy) and IRCCS Mondino. The subjects’ samples were shipped to Ospedale Fatebenefratelli ‘San Giovanni Calibita’, Isola Tiberina, Rome, Italy, for blinded biochemical analyses. 

#### 2.2.3. Biochemical Investigation Applied for the Replication Study

The aliquots were thawed just before the assay. Concentration of Cp was measured with immunoturbidimetric assay (Futura System SRL, Rome, Italy) and calibrated against the international reference preparation (ERM 470) [31], performed in duplicate on the multiple biochemical analyzer Horiba Pentra 400 (ABX Diagnostic, Montpellier, France).

The activity eCp was measured following an automation of the Schosinsky o-dianisidine eCp assay [32] adapted from our laboratory for multiple biochemical analyzers Horiba Pentra 400 (ABX Diagnostic, Montpellier, France) and described in detail elsewhere [33]. Serum Cu concentration was estimated with the colorimetric assay of Abe et al. (Randox Laboratories, Crumlin, UK) [34] and confirmed in 30% of samples by atomic absorption spectrophotometry measurements using an AAnalyst 600 (Perkin-Elmer, Norwalk, CT, USA) equipped with graphite furnace, as described in detail elsewhere [35].

Non-ceruloplasmin Cu was calculated from the equation provided by Walshe (appendix of [36]), based on the measures of concentration of total Cu and Cp in serum. Equivalent data can be obtained calculating Non-Cp Cu from mg/L of ceruloplasmin and considering the conversion of 3.15 µg/Cu for mg of ceruloplasmin [36].

The Cu:Cp [20] was calculated as reported by Twomey et al. [37]. These authors provided the following equation:

[Cu µmol/L] ∗ [132,000 (g/mol)]/[Cp (mg/dL) ∗ 10].

As we previously discussed in detail [20], the plausible theoretical values of Cu:Cp, which can be effectively measured in specimens of HC, should range between 6–8, even though this ratio can yield diverse values. A 6–8 range obtained in HC for Cu:Cp assures we obtain a more reliable non-ceruloplasmin Cu value when applying the Walshe’s formula [20,36], since Cu and ceruloplasmin are in the in the correct stoichiometry for Cu and ceruloplasmin based on the evidence that each ceruloplasmin molecule has 6–8 sites for Cu atom binding.

#### 2.2.4. Statistical Analyses Applied to the Replication Study

Continuous data were presented in terms of mean SD or, if they were not normally distributed, of median (IQR: Q1–Q3). The assumption of normality was verified using a Shapiro–Wilk test and normal Q-Q plot. Logarithmic transformation of non-Cp Cu values was applied (natural log of the absolute value of non-Cp Cu multiplied for sign of non-Cp Cu values) to reduce the error variability and better approximate normal distribution. Categorical data were presented as frequencies (percentage, %). Differences between healthy controls and AD patients in sex or haplotype AG were evaluated by a Chi-square test. Difference in age between the two diagnosis groups was tested by a *t*-test; differences in MMSE were evaluated by a nonparametric Mann–Whitney test. Differences in biochemical variables were evaluated by an ANCOVA model, adjusting for sex and age. Pearson’s correlation coefficient was calculated to quantify the relation between age and biochemical variables. 

#### 2.2.5. Procedures Applied to Run the Genetic Studies

Genomic DNA was purified from peripheral blood using the conventional method for DNA isolation (QLAamp DNA Blood Midi kit). Genotyping of rs1061472 and rs732774 was performed by the TaqMan allelic discrimination assay as described in [22]. The predesigned SNP genotyping assay IDs are ID_C_1919004_30 (rs1061472) and ID_C_938208_30 (rs732774) (Applied Biosystems, Inc, Waltham, Massachusetts, USA). DNA extraction from 4 AD patients and genotyping of additional 2 AD patients and 1 control failed.

The Quanto program showed that the study had an 85% power to detect an effect of odds ratio (OR) ≥ 2.0 for a MAF ranging between 0.440 and 0.46 considering a general (co-dominant) model, based on an alpha of 0.05 and assuming AD prevalence 5.0% in the general population.

#### 2.2.6. Statistical Analyses Applied for the Genetic Studies

Demographic and clinical characteristics in our patient sample were described either in terms of mean ± SD, if quantitative, or in terms of proportions. Student’s *t*-test and the Chi-square (χ^2^) test were used to compare the characteristics of patient and control groups using the package SPSS 21.0. 

To calculate the power of our sample, we used the Quanto 1_2_4 program (Gauderman WJ, Morrison JM 2006; http://hydra.usc.edu/gxe).

Allele, genotype, and haplotype frequencies along with the Hardy–Weinberg equilibrium were evaluated using Plink (Plink v1.07; http://pngu.mgh.harvard.edu/~purcell/plink/; accessed date 1 July 2019) and SPSS 21.0. Permutation tests were used to correct multiple testing errors with 1000 simulations. Haplotypes with frequencies greater than 5% were considered in the analyses.

## 3. Results

### 3.1. Meta-Analysis

**Figure 1:**
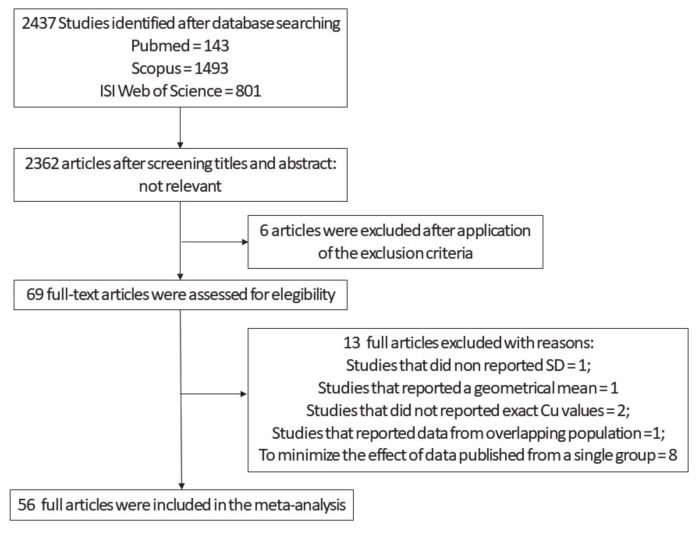
Flow diagram employed from the screening and selection of the analyzed Cu studies Table 1.

Our first selection produced an output of 2437 studies (Figure 1). After screening titles and abstracts and the exclusion of the non-relevant studies, 75 articles remained. Out of this pool, we ran an additional screening and looked for manuscripts that reported Cu values, expressed as means and SD, obtained from AD and HC subjects. Sixty-nine full-text articles were assessed for eligibility. We excluded from the original 69 one study that did not report SD [38], one that reported a geometrical mean [39], and two that did not report the absolute value of Cu levels [10,40]. We also excluded the study by Alimonti et al. [41] because the data partially overlapped with some shown in a paper by Bocca and colleagues [42]. To minimize the potential bias produced by data published from a single group, we included only a limited number of studies from ‘Fatebenefratelli research group’. Out of the 13 studies from the group, we chose five [35,43,44,45,46] since these (i) employed different methods for the analysis of Cu, ceruloplasmin, and Non-Cp Cu; (ii) employed samples from populations living in different geographical areas of the country (Italy); and (iii) were performed by three independent laboratories. On the remaining 56 studies (Figure 1, Table 1, Tables S1–S3, Supplementary Material), we carried out both study-wise and group-wise analyses. Finally, as an additional control, a supplementary analysis was performed in which all the studies produced by the Fatebenefratelli research group were pooled together and considered as a single study (Figure S1, Supplementary Material). The evaluation of the quality of the studies was assessed by the Newcastle–Ottawa Scale. The maximum number of stars was eight. The average score was equal to 5. Since there is not yet a standard cut-off, we applied arbitrary cut-off: 0–4 poor quality, 5–6 fair quality, and ≥7 high quality. On this basis, 20.5% of the studies had a high quality, 47.7% fair, and 31.8% poor.

#### 3.1.1. Meta-Analysis of Cu Data from Brain Tissues

**Figure 2:**
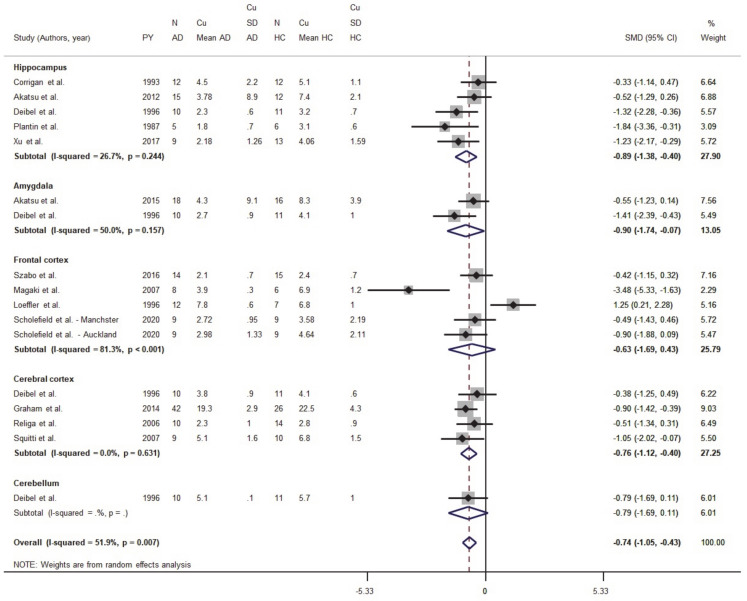
Standardized mean difference (SMD) computed from the studies on Cu brain levels (μg/g) in AD patients and HC subjects. SMDs between patients and controls are represented by squares, whose sizes are proportional to the sample size of the relative study. The whiskers represent the 95% confidence interval (CI). The diamond represents the pooled estimate based on the random-effects model, with the center representing the point estimate and the width indicating the associated 95% CI.

Eighteen studies were identified in the primary screening; after the application of the exclusion criteria described above, six were excluded [59,60,61,62,63,64]. Clinical data, demographic information limited to age, sex, ethnicity, and cerebral area of the metal measurements were obtained from 182 AD and 166 HC subjects and described in Table 1. Cu concentrations in AD and HC subjects were analyzed and regionally stratified to investigate changes in the hippocampus (five studies), amygdala (two studies), the whole cortex (four studies), the frontal cortex (five studies), and the cerebellum (one study) [50] (Figure 2). The standardized mean difference (SMD) differed and was equal to −0.74 (95% CI −1.05, −0.43; *p* < 0.001), thereby indicating that Cu levels were depleted in most regions of the brain in the AD group (Figure 2).

We also analyzed data from five studies focused on the hippocampus (Table 1) from a pool of 51 AD and 54 HC subjects. These studies reported normal or lower Cu levels in AD patients when compared to HC. The pooled SMD was = −0.89 (95% CI −1.38, −0.399; *p* < 0.001), while the heterogeneity was I^2^ = 26.7% (*p* = 0.244). 

The amygdala was considered only in two studies for a total of 28 AD and 27 HC subjects (Table 1). One study reported lower Cu concentrations in AD patients when compared to HC [50]; in this group, the pooled SMD was equal to −0.90 (95% CI −1.74, −0.07; *p* = 0.033). The heterogeneity was moderate (I^2^ = 50%, *p* = 0.157). 

Five studies reported data collected from the frontal cortex of 52 AD and 46 HC subjects. While a study by Magaki and colleagues [53] reported lower Cu levels in AD brains, a paper by Loeffler and colleagues indicated Cu increases in brain samples from AD patients [49]. However, when the authors normalized Cu concentrations to the tissue protein content, Cu levels resulted lower in AD when compared to HC subjects. In this subset, the pooled SMD was = −0.63 (95% CI −1.69, 0.43; *p* = 0.245) and an elevate heterogeneity was observed (I^2^ = 81.3%, *p* < 0.001).

Data on the whole cerebral cortex were obtained from four studies (Table 1). All these studies reported lower Cu levels in AD patients when compared to HC subjects. However, the difference was evident only in two studies [51,55] with a pooled SMD equal to −0.76 (95% CI −1.12, −0.40; *p* < 0.001) and a heterogeneity I^2^ = 0% (*p* = 0.631).

#### 3.1.2. Meta-Analysis of Cu in Serum/Plasma

**Figure 3:**
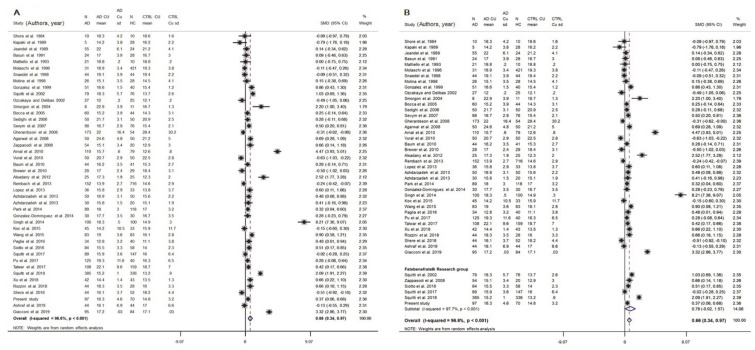
Standardized mean difference (SMD) computed from the studies on Cu serum/plasma levels (μmol/L) in AD patients and HC subjects. SMDs between patients and controls are represented by squares, whose sizes are proportional to the sample size of the relative study. The whiskers represent the 95% confidence interval (CI). The diamond represents the pooled estimate based on the random-effects model, with the center representing the point estimate and the width indicating the associated 95% CI. In panel (**A**) is the study-wise analysis; in panel (**B**) is the group-wise analysis. Abbreviations: PY, publication year; N, number; SD, standard deviation; HC, healthy controls.

Thirty-five studies on serum Cu and an additional nine studies on plasma were selected (Figure 3 and Tables S1–S3, Supplementary Material).

The analysis was performed initially considering studies on serum and plasma separately.

The results on serum samples showed significantly higher Cu values in AD patients than in HC (SMD = 0.54; 95% CI 0.23, 0.85; *p* = 0.001) and a high heterogeneity (I2 = 95.8%; *p* < 0.001). In fact, 17 studies reported higher value in AD patients than in HC [35,44,45,46,65,66,67,68,69,70,71,72,73,74,75,76] and two studies showed lower Cu values in AD than in HC [77,78], while the remaining studies reported no significant results.

The results on plasma samples showed no significant difference (SMD = 1.11; 95% CI−0.06, 2.27; *p* = 0.062) and a high heterogeneity (I^2^ = 98.2%; *p* < 0.001). In fact, three studies reported no significant results [79,80,81]. One study reported higher Cu values in AD patients than in HC [82,83,84,85] and two showed lower Cu values in AD than in HC [86,87].

We then carried out a comprehensive meta-analysis including both serum and plasma samples. Data came from 2929 AD patients and 3547 healthy controls (46 studies). Patient samples ranged from 5 to 385 individuals. The mean age of the participants was >70 years, except for a few studies that investigated younger subjects (Table S1, Supplementary Material). The percentage of women in the AD group ranged from 20% to 100%. The analyzed population was Caucasian in 20 studies, Asian in 17, and Argentinean in 1 study. Both study-wise and group-wise analyses showed higher serum/plasma Cu levels in AD patients when compared to HC subjects (SMD = 0.66, 95% CI 0.34, 0.97, *p* < 0.001; Figure 3). A supplementary analysis in which all studies carried out by ‘Fatebenefratelli research’ group were pooled together and taken into account as a single study, confirmed higher serum/plasma Cu levels in AD patients when compared to HC subjects (SMD = 0.64, 95% CI 0.31, 0.93, *p* < 0.001; Figure S1, Supplementary Material). Heterogeneity was high (I2 = 95.9%; *p* < 0.001).

The funnel plot (Figure S2, Supplementary Material) appeared asymmetrical, but asymmetry was not relevant (Egger test: beta = 1.30, SE = 2.21; *p* = 0.560). The meta-regression revealed that the difference in the mean age between AD and HC subjects did not affect the analysis (beta = −0.01, SE = 0.019; *p* = 0.714). The difference of the SMD for Cu in serum/plasma was 0.66 and therefore the formula,

(1) $$SMD=\frac{\surd 3}{\pi }\;\surd lnOR,$$

delivered an OR = 3.30 (95% CI 1.86, 5.85), indicating that a µmol/L unit increase in serum/plasma Cu resulted in fourfold higher odds in AD patients than in HC individuals. 

#### 3.1.3. Meta-Analysis of Non-Cp Cu

**Figure 4:**
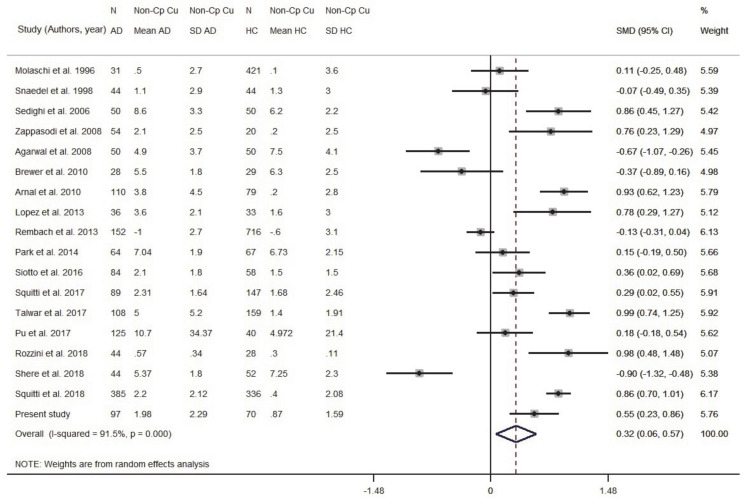
Standardized mean difference (SMD) computed from the studies on Non-Cp Cu(µmol/L) in AD patients and HC subjects. SMDs between AD subjects and controls are represented by squares, whose sizes are proportional to the sample size of the relative study. The whiskers represent the 95% confidence interval (CI). The diamond represents the pooled estimate based on the random-effects model, with the center representing the point estimate and the width indicating the associated 95% CI. Abbreviations: PY, publication year; N, number; SD, standard deviation; HC, healthy controls.

Eighteen studies were taken into consideration (Figure S4 and Table S3, Supplementary Material). Data originated from 1595 AD and 2399 HC subjects. The patient sample size ranged from 28 to 385 individuals; the control sample size ranged from 20 to 716 (Table S3, Supplementary Material). Results indicated that AD subjects had higher levels of Non-Cp Cu compared to HC subjects (SMD = 0.32, 95% CI 0.06, 0.57, *p* = 0.014; Figure 4). There was a high heterogeneity among the included studies (I2 = 91.5%; *p* < 0.001). To check for possible publication bias, we performed a funnel plot (Table S5, Supplementary Material) which revealed no evidence of asymmetry, as confirmed by the Egger test (b = −1.98, SE = 2.19; *p* = 0.380). 

**Figure 5:**
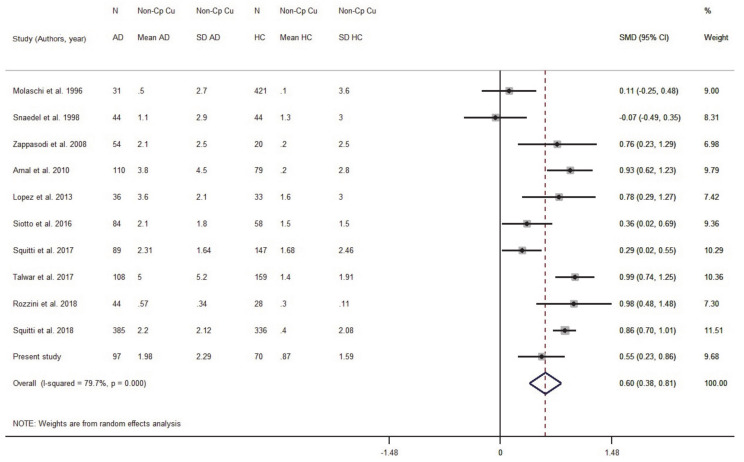
Standardized mean difference (SMD) computed from the studies on Non-Cp Cu(µmol/L) in AD patients and HC subjects when considering studies with a Cu:Cp ratio lower than 6 and higher than 8. SMDs between AD subjects and controls are represented by squares, whose sizes are proportional to the sample size of the relative study. The whiskers represent the 95% confidence interval (CI). The diamond represents the pooled estimate based on the random-effects model, with the center representing the point estimate and the width indicating the associated 95% CI. Abbreviations: PY, publication year; N, number; SD, standard deviation; HC, healthy controls.

We ran a sensitivity analysis taking into account the Cu:Cp ratio as a Cu index for internal quality control to verify the ceruloplasmin calibration. The ratio provides information about the actual stoichiometry between Cu and ceruloplasmin in the specimens. As we discussed elsewhere [20], a 6–8 Cu:Cp range in HC individuals allows us to obtain more reliable Non-Cp Cu values when applying the Walshe’s formula [20,36]. On this basis, seven studies reporting in HC subjects a Cu:Cp ratio lower than 6 and higher than 8 were excluded from the meta-analysis (Figure 5). By excluding these seven studies, the pooled AD sample was included 1082 subjects and the pooled HC sample 1289 subjects. The result was SMD = 0.59 (95% CI 0.38, 0.81; *p* < 0.001) and the heterogeneity I^2^ = 79.7% (*p* < 0.001).

The meta-regression revealed that the difference in the mean age between AD patients and HC did not affect the analysis (beta = 0.34, SE = 0.19; *p* = 0.095). The difference of the SMD for Non-Cp Cu in serum/plasma was 0.32, which delivered OR = 1.79 when applying Formula (1), indicating that for a µmol/L unit increase in Non-Cp Cu, there was a twofold increase in odds of having AD compared to HC. Considering the results of the sensitivity analysis, SMD = 0.59 gave OR = 2.91 (95% CI 1.99, −4.35; *p* < 0.001), indicating that for a µmol/L unit increase in Non-Cp Cu, there was a threefold increase in odds of having AD compared to HC.

#### 3.1.4. Meta-Analysis of Ceruloplasmin in Serum/Plasma

Seventeen studies were included in the meta-analysis (Table S3, Supplementary Material). Data were from 1551 AD patients and 2371 HC. There was no difference in ceruloplasmin levels between AD patients and healthy controls (SMD = 0.04, 95% CI −0.11, 0.19 (*p* = 0.589); Figure S4 Supplementary, Materials). A high heterogeneity among the included studies was revealed (I^2^ = 75.3%, *p* < 0.001). No publication bias was observed (beta = 0.48, SE = 1.34; *p* = 0.727; Figure S5, Supplementary Material). The meta-regression did not show a relevant effect of the difference in the mean age between AD patients and HC subjects (beta = 0.088, SE = 0.12; *p* = 0.487).

### 3.2. Replication Study of Changes in Serum Cu Biomarkers in AD and HC Subjects

A total of 167 participants were recruited for this study (97 AD and 70 HC; Table 2). While ceruloplasmin did not correlate with age, a slightly positive association between age and specific activity of ceruloplasmin (r = 0.244; *p* = 0.002), Non-Cp Cu (r = 0.311; *p* < 0.001) and Cu:Cp ratio (r = 0.28; *p* < 0.001) was observed (Table 2). 

The two study groups were no different for sex distribution, even though men were prominent in the AD group (HC men: 22.9% (16/70) vs. AD men: 37.1% (36/97); Chi square = 3.85, df (1) *p* = 0.050), and Cu, ceruloplasmin levels and activity were found to be different between men and women. To correct for this potential bias, all the analyses were corrected for sex and age. In HC subjects, the correlation between ceruloplasmin concentrations and serum Cu levels was 0.86 (*p* < 0.001), the correlation between ceruloplasmin concentrations and ceruloplasmin activity values was 0.75 (*p* < 0.001), the correlation between ceruloplasmin activity and serum Cu levels was 0.77 (*p* < 0.001), and between Non-Cp Cu and the Cu:Cp ratio was 0.97 (*p* < 0.001). The Cu:Cp ratio in HC was 6.6 (0.81), thereby demonstrating a good agreement between Cu and ceruloplasmin, allowing the application of Walshe’s formula [36]. Among the variables under study, total serum Cu, Non-Cp Cu, and the Cu:Cp ratio were higher in AD patients (Table 2), and 44% of AD patients had values of Non-Cp Cu higher than 1.6 µmol/L (upper limit of normal) [36,88,89]. 

The genetic study analyzed the allele, genotype, and haplotype distribution of *ATP7B* rs732774 and rs1061472 in association with the main demographic and clinical characteristics of 91 AD patients and 69 HC (genotype data from six AD patients and one HC were not available; Table 3). A difference between AD patients and HC was found when analyzing genotype frequencies for rs732774 (adjusted *p-*values = 0.032). As for allele carrier frequencies (AA + AG), carriers of at least one A allele were more frequent in the AD group compared to HC (92% vs.78%; adjusted *p*-values = 0.011, OR = 3.33, 95% CI 1.28, 8.70; Table 3). 

As for rs1061472, no differences emerged between the two study groups (adjusted *p-*values = 0.097), while carriers of at least one G allele (GG + GA) were more frequent in the patient group (89% vs. 75%; adjusted *p-*values = 0.031, OR = 2.65, 95% CI 1.13, 6.21).

Finally, we built the haplotypes of the two polymorphisms and observed that cluster genotype frequencies were different between AD and HC (adjusted *p-*values = 0.022); in particular, AG haplotype carriers were more frequent in the AD group (89% vs. 75%; adjusted *p-*values = 0.012, OR = 3.30, 95% CI 1.26, 8.69).

A univariable binary logistic regression model was applied to evaluate the effect of biochemical variables on the probability of having AD. The model also took into consideration AG haplotype, age, and sex (Table 4). A multivariable analysis was performed that included the biochemical variables with a *p-*value < 0.10 at the univariable analysis. The model revealed an effect of Non-Cp Cu on the probability of having AD with an increased risk of 1.32 times (95% CI 1.06, 1.64; *p* = 0.012) for each µmol/L unit increase in Non-Cp Cu when keeping all other independent variables constant. In the multivariable model, the effect of the AG haplotype on the probability of having AD was less consistent (95% CI 0.99, 6.28; *p* = 0.053) with respect to the result obtained in the univariable model (OR = 2.7 95% CI 1.13, 6.23, *p* = 0.026), with a probability of having AD that was about 2.49 times higher in the individual carriers of the AG haplotype compared to the GA/GA haplotype carriers, likely due to the limited number (only 27) of individual carriers of the GA/GA haplotype; Table 3).

## 4. Discussion

We have conducted a quantitative meta-analysis, using non-overlapping data from approximately 6991 participants from 56 studies, to help clarify available evidence on the levels of Cu in AD. Overall, our results indicate that Cu decreases in AD brain specimens (pooled total of 182 AD and 166 healthy controls HC), that Cu (pooled total of 2929 AD and 3547 HC) and Non-Cp Cu (pooled total of 1595 AD and 2399 HC) increase in serum/plasma samples, and that ceruloplasmin does not change. Circulating Cu excess is associated with a three to fourfold increase in the risk of having AD. The replication study confirms meta-analysis results and shows that carriers of the AG haplotype of the *ATP7B* gene are significantly more frequent in the AD group. As a whole, these data provide a concise and organic picture of copper imbalance in AD that can allow a new interpretation of the role played by the metal in the disease: that brain Cu decreases coexisting with circulating Cu increases mirror the processes that take place in WD, another condition associated with neurodegenerative processes and Cu dysregulation. WD is characterized by the presence of higher than normal Non-Cp Cu values, a change that is an established WD biomarker [88,89]. Furthermore, in the early stages, WD is characterized by normal or even low levels of Cu in the brain, as reported in some studies on the toxic milk mouse [90] and on the Long Evans Cinnamon rat, two preclinical WD models [91]. In line with the analogy between the two diseases, functional SNPs of *ATP7B* [23], also known as the WD gene, have been associated with increased susceptibility to AD in a subset of patients [92], in line with current results on the distribution of the rs732774 and rs1061472 *ATP7B* AG haplotype. Thus, current meta-analysis shows that some distinct, Cu-related, molecular changes that have hitherto been considered peculiar to WD are also present in AD patients, thereby providing biological plausibility to our results [12,20]. 

The analysis of data obtained from brain specimens shows a regional Cu imbalance that affects the hippocampus, amygdala, frontal cortex, and several additional areas of the cerebral cortex. The data reveal a high heterogeneity that was present particularly in the frontal cortex and is likely due to differences in the magnitude of the effect size and the methodology used to assess Cu in each study. 

The meta-analysis investigating differences in Cu and Non-Cp Cu levels in serum/plasma indicate increased levels of Cu and reveal a high degree of heterogeneity. 

We checked for heterogeneity due to differences in the methods applied to assess Cu concentrations in serum/plasma and four “classes of method” were identified: Atomic absorption spectroscopy (AAS, 14 serum studies; 3 plasma studies), colorimetric methods (12 serum studies), inductively coupled plasma mass spectrometry (ICP-MS, 9 serum studies; 4 plasma studies), and energy dispersive X-ray fluorescence (EDXRF, 2 plasma studies). The analysis revealed that the heterogeneity could not be explained by difference in the methodology applied to Cu assessment. Furthermore, within each class of method, a high degree of heterogeneity was observed. 

However, a more intriguing explanation for heterogeneity in line with the results of the replication study can be built taking into consideration that the data suggest the presence of a skewed percentage of AD subjects who exhibit increased Non-Cp Cu levels (higher than 1.6 µmol/L, the upper limit of normal) [36,88,89], thereby indicating a subset of AD subjects who are particularly prone to Cu dysmetabolism (reviewed in [9]). The presence of this Cu-related subset of AD patients with Non-Cp Cu values higher than 1.6 µmol/L can explain the increased levels of serum/plasma Cu and Non-Cp Cu found in the meta-analysis. Moreover, previous studies have demonstrated increased levels of Non-Cp-Cu in 50% of AD patients (reviewed in [9]). The use of Non-Cp Cu as an AD biomarker has a 95% specificity but, unfortunately, only a 40–50% sensitivity [43]. Therefore, Non-Cp Cu is not suitable as a diagnostic biomarker for the disease. Nevertheless, Non-Cp Cu has prognostic value and can be helpful in intercepting the conversion from Mild Cognitive Impairment (MCI) to symptomatic AD in a subset of patients who exhibit peripheral signs of Cu imbalance [93]. Thus, Non-Cp Cu levels can be employed as an inclusion and stratification criterion for eligibility assessment in early phase II clinical trials testing anti-Cu therapy.

Of high clinical relevance, taking into account the SMD resulting from the current study, our data reveal that the probability of having AD increases four times for each µmol/L unit increment of peripheral Cu and three times for each µmol/L unit increase in peripheral Non-Cp Cu, in line with previous retrospective [94] and prospective studies [93,95]. 

Results of the replication study confirm the meta-analysis outcomes, revealing increased Non-Cp Cu levels in a new independent population of AD patients. The genetic study, with a power of 85%, showed that A rs732774 and G rs1061472 and the AG *ATP7B* haplotype were more frequent in our AD patients. The multivariable model built on these data indicates that Non-Cp Cu was associated with a 1.32-fold increased risk for AD for each µmol/L unit increase. The *ATP7B* AG haplotype was associated with an increased risk of having AD in the univariable model. These results should be taken with caution as the small number of GA/GA carriers reduced the strength of the association when computed in the multivariable model (Table 4). In a final analysis, Non-Cp Cu is determined to be the strongest factor associated with the probability of having AD.

What are the biological processes underlying these intriguing results? Non-Cp Cu is a low molecular weight pool of exchangeable Cu compounds circulating in the bloodstream that easily crosses the BBB and becomes toxic when exceeding 1.6 µmol/L [36,88], fueling the labile Cu reservoir in the brain. A consistent number of in vivo studies in preclinical AD models demonstrated that small amounts (around 0.13 ppm) of Cu ingested through drinking water can double Non-Cp Cu levels, reduce the cerebrospinal fluid clearance of Aβ [96] increase levels of Cu in the hippocampus, increase Aβ production, enhance oxidative stress, and generate behavioral and cognitive deficits in diverse experimental settings (recently reviewed in [97]). AβPP/Aβ are Cu/Zn binding proteins with dual role as potential natural Cu/Zn buffering proteins or as toxic compounds due to overload of metal-Aβ [12]. We recently proposed a new model that can explain how Cu misplacement and dysregulation might accelerate the onset and progression of AD [12]. According to the model, the AD Cu misbalance can be described by the presence of a single control variable—a critical, location-dependent, Cu dissociation constant, K_dc_ [12]. The loss of functional Cu from protein-bound pools is key to decreased energy production and impaired oxidative stress control. The loss of functional Cu from protein-bound pools is characterized by a reduced pool of divalent Cu(II) with K_d_ < K_dc_. The gain of redox-toxic function can be described as the existence of more Cu with K_d_ > K_dc_. In serum/plasma, a K_dc_ ~ 10^−12^ M is estimated as the critical threshold, whereas at synapses, the K_dc_ value could be ~10^−9^ M. At synapses, the threshold is close to K_d_ values for Cu(II)-binding to Aβ, prion protein, APP, and α-synuclein [12]. The data of the current meta-analysis support the proposed model based on an altered Cu K_dc_ present in AD. The shift toward the build-up of an increased pool of loosely bound Cu can facilitate the activation of Cu-driven bioenergetic abnormalities in mitochondria, impair glucose utilization, increase oxidative stress, inflammation, calcium dyshomeostasis, and interfere with the balance of other brain metals such as Fe and Zn [12]. The altered Cu availability also has relevant synaptic effects by interfering with critical proteins that are involved in Cu buffering and functioning of glutamatergic synapses [12]. These processes might facilitate the aggregation of Aβ oligomers in insoluble plaques and favor the formation of amyloid plaques short-circuiting the neuronal networks to which those synapses belong. Cu also affects the function and structure of crucial Cu proteins such as prion proteins, α-synuclein, cytochrome C oxidase, SOD1, ceruloplasmin, ATPase7B, metallothioneins, and dopamine beta-hydroxylase [12], and can drive the intracellular accumulation of phosphorylated Tau due to the presence of Cu-binding sites on the protein [98]. 

Evidence of the role of Cu in AD collected so far, along with data presented in current study, can substantiate the merit of evidence-based medicine to guide decision-making in prevention of AD with regard to Cu dysfunction. The report on Drinking Water of 2000 [99] refers that heterozygote carriers of *ATP7B* mutations for WD represent 1% of general population and that these people might have some abnormalities in Cu regulation in relation to Cu leaching from drinking water pipelines. Consistently, as presented in the current study, individuals who are carriers of the G allele in rs1061472 and the A allele in rs732774 have increased levels of Non-Cp Cu in general circulation and other Cu abnormalities linked to an increased risk of AD. These considerations are suggestive of the fact that carriers of WD *ATP7B* heterozygosity or carriers of AD related *ATP7B* SNPs might be sensitive to Cu exposure and be at risk for developing dementia. We feel that the theoretical framework of Cu imbalance in AD together with current evidence demonstrating that a body Cu dysfunction contributes to increasing the risk of AD is robust and can justify dietary and lifestyle changes to reduce this risk. Implementing a low Cu diet to regain Cu balance or, if using multiple vitamin supplements, to choose those without Cu, as per expert committees’ suggestions [100], might be simple and practical actions to take in the attempt to decrease the chance to developing AD when Cu imbalance has been assessed, which can be easily carried out by means of measuring serum non-Cp Cu excess (values higher than 1.6 µmol/L) [100,101]. This recommendation is based on the fact that lifestyle and dietary changes may be justified not only in cases in which available evidence proves risk beyond any doubt, but even in cases in which evidence of risk is substantial. Nevertheless, as per the established modifiable risk factors, reducing the risk does not necessarily mean prevention, and individuals who adopt dietary changes to reduce their risk may still develop dementia, but have lower chances with respect to those who had not taken any measures.

However, we believe that the ultimate proof that can elucidate if Cu plays a causative role in AD would be a Phase II clinical trial set at testing therapeutic strategies aimed at counteracting Cu misbalance. In that regard, we are involved in an ongoing phase II clinical trial (PTC-19-60232) that employs a zinc-based therapy on MCI individuals who show signs of Cu dysregulation (Non-Cp Cu levels > 1.6 µmol).

The current study has several limitations: (i) case-control investigations might be affected by varying levels of bias owing to the quality of study evaluation; (ii) the heterogeneity was a problem that may have affected the precision of the overall results in this meta-analysis; (iii) a potential sampling bias as most of the studies adopted the NINCDS-ADRDA criteria [27,102] that are less stringent than the currently employed IGW-2 criteria [28]; and (iv) the lack of *APOE* genotypes. Furthermore, (v) the study did not analyze data of Cu biomarkers in cerebrospinal (CSF), because the data was too extensive to be reported in this concise presentation, even though a recent investigation reported that CSF ceruloplasmin levels predict cognitive decline and brain atrophy in people with underlying Aβ pathology [103]. Finally, (vi) the current study has not addressed the causation issue.

## 5. Conclusions

Notwithstanding its limitations, the meta-analysis results appear robust, since they have been based on a high number of studies, most with big sample sizes, and low variability in the composition of the control groups. As a whole, our results reinforce the often under-recognized importance of Cu imbalance in AD. This investigation provides data that may help to modify the general take on Cu involvement in the disease by the AD community and hopefully overcome controversy about the role played by this metal in AD. Furthermore, the study proposes that Cu dyshomeostasis could be considered as an AD susceptibility factor and that it is mainly present in a subset of AD patients who can benefit from precision-medicine therapeutic strategies set at targeting this phenomenon.

## Figures and Tables

**Table 1:** Studies included in the meta-analysis of brain Cu in Alzheimer’s dementia.

Study (Authors, Year)	Country	Brain Region	Meta-Analysis Classification	Alzheimer’s Dementia				Healthy Controls			
				N	Sex (F)	Mean Age (SD)	Cu μg/g (SD)	N	Sex (F)	Mean Age (SD)	Cu μg/g (SD)
Plantin et al., 1987 [47]	Sweden	temporal lobe	hippocampus	5			1.8 (0.7)	6			3.1 (0.6)
Corrigan et al., 1993 [48]	UK	hippocampal tissue	hippocampus	12	10	79.5 (9.2)	4.5 (2.2)	12	4	78.5 (9.0)	5.1 (1.1)
Loeffler et al., 1996 [49]	USA	frontal cortex	frontal cortex	12		79.4 (1.7)	7.8 (0.6)	7		75.7 (2.8)	6.8 (1.0)
		Amygdala	Amygdala	10	3	80.4	2.7 (0.9)	11	8	81.7	4.1 (1.0)
		Hippocampus	hippocampus				2.3 (0.6)				3.2 (0.7)
Deibel et al., 1996 [50]	USA	superior and middle temporal	cerebral cortex				3.2 (0.6)				4.0 (0.6)
		inferior parietal	cerebral cortex				3.8 (0.9)				4.1 (0.6)
		Cerebellum	Cerebellum				5.1 (0.1)				5.7 (1.0)
Squitti et al., 2007 [51]	Italian	cortical tissue	cerebral cortex	9		84.6	5.1 (1.6)	10		80.2 (6.8)	6.8 (1.5)
Religa et al., 2006 [52]	Australia	neocortical tissue	cerebral cortex	10	7	81.6 (11)	2.3 (1.0)	14	12	82.8 (11.2)	2.8 (0.9)
Magaki et al., 2007 [53]	USA	frontal cortex	frontal cortex	8	6	78 (12)	3.9 (0.3)	6	2	72 (11.0)	6.9 (1.2)
Akatsu et al., 2012 [54]	Japan	Hippocampus	hippocampus	15	9	87.5 (7.6)	3.78 (8.9)	12	6	84 (6.7)	7.4 (2.1)
		Amygdala	Amygdala	18	11		4.3 (9.1)	16	10	85.9 (7.3)	8.3 (3.9)
Graham et al., 2014 [55]	UK	Brodman area 7	cerebral cortex	42	21	83 (7.2)	19.3 (2.9)	26	13	81.7 (6.2)	22.5 (4.3)
Szabo et al., 2016 [56]	USA	frontal cortex	frontal cortex	14		78	2.1 (0.7)	15		88	2.4 (0.7)
Xu et al., 2017 [57]	New Zealand	Hippocampus	hippocampus	9	4	72 (60–80)	2.18 (1.26)	13	5	73 (61–78)	4.06 (1.59)
Scholefield et al., 2020 [58]	UK	Cingulate gyrus	frontal cortex	9	6	83 (61–89)	2.72 (0.95)	9	3	89 (82–95)	3.58 (2.19)
Scholefield et al., 2020 [58]	New Zealand	Cingulate gyrus	frontal cortex	9	4	72 (60–80)	2.98 (1.33)	9	2	73 (61–78)	4.64 (2.11)

**Table 2:** Demographics and biological variables of participants to the replication study investigating variations of Cu markers in the serum of AD subjects and healthy controls.

		Alzheimer’s Dementia	Healthy Controls	Statistics	*p*-Value	
		*N* = 97	*N* = 70		
Demographic variables						
Sex, M	% (n/subjects)	37.1% (36/97)	22.9% (16/70)		0.05	
Age	mean (SD)	71.1 (7.18)	65.9 (7.53)		0.01	
MMSE	median (Q1-Q3)	19 (15–23)	29 (27.3–30)		<0.001 *	
Biological variables						
Cu (µmol/L)	mean (SD)	15.8 (3.76)	14.6 (2.95)	F(1, 163) = 6.69 ^#^	0.011	
Ceruloplasmin (mg/dL)	mean (SD)	29.3 (4.89)	29.2 (4.08)	F(1, 163) = 1.13 ^#^	0.289	
Non-ceruloplasmin Cu *^,&,†^ (µmol/L)	median(Q1-Q3)	1.4 (0.6–2.8)	0.75 (−0.16–1.71)	F(1, 163) = 9.88 ^#^	0.002	
Ceruloplasmin activity (IU)	mean (SD)	117.9 (26.13)	110.4 (18.06)	F(1, 163) = 10.21 ^#^	0.008	
eCp:iCp ratio	mean (SD)	4.0 (0.51)	3.8 (0.44)	F(1, 163) = 7.02 ^#^	0.009	
Cu:Cp ratio	mean (SD)	7.1 (0.74)	6.6 (0.93)	F(1, 163) = 10.21 ^#^	0.002	

**^#^** All analyses were adjusted for sex and age; * non-parametric Mann–Whitney test; ^&^ logarithmic transformation was applied as described in method section; ^†^ mean (SD) value of Non-Cp Cu in AD patients was 2 (2.29) µmol/L and that in HC was 0.9 (1.59) µmol/L [F(1, 163) = 11.23, *p* = 0.001].

**Table 3:** Genetic characteristics of the investigated AD patients and HC subjects.

	Healthy Controls		AD		Chi-Square				Odd Ratio		
rs732774	*n*	Freq	*n*	Freq	Value	df	*p*	p_correct_	Value	95% CI	
**Alleles**											
A	78	0.57	113	0.62							
G	60	0.43	69	0.38	1.011	1	0.315	0.409			
Total	138	1.00	182	1.00							
**Genotypes**											
AA	24	0.35	29	0.32							
AG	30	0.43	55	0.60	7.857	2	0.020	0.032			
GG	15	0.22	7	0.08							
Total	69	1.00	91	1.00							
**Carriers**											
Allele A	54	0.78	84	0.92	6.529	1	0.011	0.011	3.33	1.28	8.70
Allele G	45	0.65	62	0.68	0.151	1	0.698	0.799	1.14	0.59	2.21
	**Healthy Controls**		**AD**		**Chi-Square**				**Odd Ratio**		
**rs1061472**	***N***	**Freq**	***N***	**Freq**	**Value**	**df**	***p***	**p_correct_**	**Value**	**95% CI**	
**Alleles**											
A	64	0.46	72	0.40							
G	74	0.54	110	0.60	1.492	1	0.222	0.315			
Total	138	1.00	182	1.00							
**Genotypes**											
AA	17	0.25	10	0.11							
AG	30	0.43	52	0.57	5.762	2	0.056	0.097			
GG	22	0.32	29	0.32							
Total	69	1.00	92	1.00							
**Carriers**											
Allele A	47	0.68	62	0.68	4.6 × 10^−6^	1	0.998	1.000	1.00	0.51	1.96
Allele G	52	0.75	81	0.89	5.212	1	0.022	0.031	2.65	1.13	6.21

n subjects in the analysis = 160; AD = 91, healthy controls = 69.

**Table 4:** Results of a uni- and a multivariable binary logistic model developed to evaluate the effect of molecular variables on the probability of having AD.

	Univariable Analysis			Multivariable Analysis *		
	OR	95% CI	*p*-Value	OR	95% CI	*p*-Value
**Sex (M vs. F)**	1.99	0.99–3.98	**0.051**	2.51	1.13–5.56	0.023
**Age**	1.06	1.01–1.1	**0.008**	1.03	0.98–1.08	0.193
**Cu**	1.11	1.01–1.22	**0.037**			
**iCp**	1.01	0.94–1.08	0.845			
**eCp**	1.02	1.0–1.032	**0.045**			
**Non-Cp Cu**	1.4	1.14–1.70	**0.001**	1.32	1.06–1.64	0.012
**eCp:iCp ratio**	2.86	1.36–6.0	**0.006**	2.17	0.96–4.91	0.062
**Cu:Cp ratio**	1.17	0.46–3.01	0.74			
Haplotypes *						
AG vs. GA/GA	2.7	1.13–6.23	0.026	2.49	0.99–6.28	0.053

Table notes: Biochemical variables with a *p-*value < 0.10 at the univariable analysis (bold values in the table) were included in the multivariable analysis. Cu and eCp were not included to avoid the multicollinearity with Non-Cp Cu and eCp:iCp ratio, respectively. * The analysis was carried out on 160 individuals. OR, odds ratio; CI, confidential Interval.

## Data Availability

Data supporting reported results are provided in Supplementary Materials and can be provided by contacting the corresponding authors.

## Supplementary Materials

The following are available online at https://www.mdpi.com/article/10.3390/biom11070960/s1. Table S1: Demographic data of the eligible studies for meta-analysis of copper markers in serum/plasma; Table S2: Serum or plasma Cu levels in Alzheimer’s dementia patients and in healthy controls; Table S3: Indices of serum Cu status in all the 18 studies employed for the meta-analysis of non-Ceruloplasmin Cu (Non-Cp Cu) and ceruloplasmin (Cp); Figure S1: Standardized mean difference (SMD) computed from the studies on Cu serum/plasma levels (μmol/L) in AD patients and HC subjects; Figure S2: Funnel plot suggested no presence of publication bias in the studies of Cu in serum/plasma evaluated in Alzheimer’s dementia and Healthy Controls; Figure S3. Funnel plot suggested no presence of publication bias in the studies of non-ceruloplasmin Cu in serum/plasma evaluated in Alzheimer’s dementia and Healthy Controls; Figure S4. Standardized mean difference (SMD) computed from the studies on ceruloplasmin (mg/dL) in Alzheimer’s dementia patients and Healthy Controls; Figure S5. Funnel plot suggested no presence of publication bias in the studies of ceruloplasmin in serum/plasma evaluated in Alzheimer’s disease and healthy controls.

## References

[1] Alzheimer’s-Association (2019). 2019 Alzheimer’s disease facts and figures. Alzheimer’s Dement..

[2] Kepp K.P. (2017). Ten Challenges of the Amyloid Hypothesis of Alzheimer’s Disease. J. Alzheimer’s Dis..

[3] Herrup K. (2015). The case for rejecting the amyloid cascade hypothesis. Nat. Neurosci..

[4] Hardy J., Selkoe D.J. (2002). The amyloid hypothesis of Alzheimer’s disease: Progress and problems on the road to therapeutics. Science.

[5] Masters C.L., Selkoe D.J. (2012). Biochemistry of amyloid beta-protein and amyloid deposits in Alzheimer disease. Cold Spring Harb. Perspect. Med..

[6] Norton S., Matthews F.E., Barnes D.E., Yaffe K., Brayne C. (2014). Potential for primary prevention of Alzheimer’s disease: An analysis of population-based data. Lancet Neurol..

[7] Barnes D.E., Yaffe K. (2011). The projected effect of risk factor reduction on Alzheimer’s disease prevalence. Lancet Neurol..

[8] Bush A.I., Tanzi R.E. (2008). Therapeutics for Alzheimer’s disease based on the metal hypothesis. Neurotherapeutics.

[9] Sensi S.L., Granzotto A., Siotto M., Squitti R. (2018). Copper and Zinc Dysregulation in Alzheimer’s Disease. Trends Pharmacol. Sci..

[10] James S.A., Volitakis I., Adlard P.A., Duce J.A., Masters C.L., Cherny R.A., Bush A.I. (2012). Elevated labile Cu is associated with oxidative pathology in Alzheimer disease. Free Radic. Biol. Med..

[11] Cheignon C., Tomas M., Bonnefont-Rousselot D., Faller P., Hureau C., Collin F. (2018). Oxidative stress and the amyloid beta peptide in Alzheimer’s disease. Redox Biol..

[12] Kepp K.P., Squitti R. (2019). Copper imbalance in Alzheimer’s disease: Convergence of the chemistry and the clinic. Coord. Chem. Rev..

[13] Faller P., Hureau C., la Penna G. (2014). Metal ions and intrinsically disordered proteins and peptides: From Cu/Zn amyloid-beta to general principles. Acc. Chem. Res..

[14] Hsu H.W., Rodriguez-Ortiz C.J., Lim S.L., Zumkehr J., Kilian J.G., Vidal J., Kitazawa M. (2019). Copper-Induced Upregulation of MicroRNAs Directs the Suppression of Endothelial LRP1 in Alzheimer’s Disease Model. Toxicol. Sci..

[15] Ma Q., Ying M., Sui X., Zhang H., Huang H., Yang L., Huang X., Zhuang Z., Liu J., Yang X. (2015). Chronic copper exposure causes spatial memory impairment, selective loss of hippocampal synaptic proteins, and activation of PKR/eIF2alpha pathway in mice. J. Alzheimer’s Dis..

[16] Wu M., Han F., Gong W., Feng L., Han J. (2016). The effect of copper from water and food: Changes of serum nonceruloplasmin copper and brain’s amyloid-beta in mice. Food Funct..

[17] Yu J., Luo X., Xu H., Ma Q., Yuan J., Li X., Chang R.C., Qu Z., Huang X., Zhuang Z. (2015). Identification of the key molecules involved in chronic copper exposure-aggravated memory impairment in transgenic mice of Alzheimer’s disease using proteomic analysis. J. Alzheimer’s Dis..

[18] Schrag M., Mueller C., Oyoyo U., Smith M.A., Kirsch W.M. (2011). Iron, zinc and copper in the Alzheimer’s disease brain: A quantitative meta-analysis. Some insight on the influence of citation bias on scientific opinion. Prog. Neurobiol..

[19] Li D.-D., Zhang W., Wang Z.-Y., Zhao P. (2017). Serum Copper, Zinc, and Iron Levels in Patients with Alzheimer’s Disease: A Meta-Analysis of Case-Control Studies. Front. Aging Neurosci..

[20] Squitti R., Simonelli I., Ventriglia M., Siotto M., Pasqualetti P., Rembach A., Doecke J., Bush A.I. (2014). Meta-analysis of serum non-ceruloplasmin copper in Alzheimer’s disease. J Alzheimers Dis..

[21] Schrag M., Mueller C., Zabel M., Crofton A., Kirsch W.M., Ghribi O., Squitti R., Perry G. (2013). Oxidative stress in blood in Alzheimer’s disease and mild cognitive impairment: A meta-analysis. Neurobiol. Dis..

[22] Squitti R., Polimanti R., Bucossi S., Ventriglia M., Mariani S., Manfellotto D., Vernieri F., Cassetta E., Ursini F., Rossini P.M. (2013). Linkage disequilibrium and haplotype analysis of the ATP7B gene in Alzheimer’s disease. Rejuvenation Res..

[23] McCann C.J., Jayakanthan S., Siotto M., Yang N., Osipova M., Squitti R., Lutsenko S. (2019). Single nucleotide polymorphisms in the human ATP7B gene modify the properties of the ATP7B protein. Metallomics.

[24] Hozo S.P., Djulbegovic B., Hozo I. (2005). Estimating the mean and variance from the median, range, and the size of a sample. BMC Med. Res. Methodol..

[25] Wan X., Wang W., Liu J., Tong T. (2014). Estimating the sample mean and standard deviation from the sample size, median, range and/or interquartile range. BMC Med. Res. Methodol..

[26] Higgins J., Thomas J., Chandler J., Cumpston M., Li T., Page M., Welch V.A. (2019). Cochrane Handbook for Systematic Reviews of Interventions Version 6.0.

[27] Dubois B., Feldman H.H., Jacova C., Dekosky S.T., Barberger-Gateau P., Cummings J., Delacourte A., Galasko D., Gauthier S., Jicha G. (2007). Research criteria for the diagnosis of Alzheimer’s disease: Revising the NINCDS-ADRDA criteria. Lancet Neurol..

[28] Dubois B., Feldman H.H., Jacova C., Hampel H., Molinuevo J.L., Blennow K., DeKosky S.T., Gauthier S., Selkoe D., Bateman R. (2014). Cummings, Advancing research diagnostic criteria for Alzheimer’s disease: The IWG-2 criteria. Lancet Neurol..

[29] Folstein M.F., Folstein S.E., McHugh P.R. (1975). “Mini-mental state”. A practical method for grading the cognitive state of patients for the clinician. J. Psychiatr. Res..

[30] Squitti R., Pasqualetti P., Forno G.D., Moffa F., Cassetta E., Lupoi D., Vernieri F., Rossi L., Baldassini M., Rossini P.M. (2005). Excess of serum copper not related to ceruloplasmin in Alzheimer disease. Neurology.

[31] Wolf P.L. (1982). Ceruloplasmin: Methods and clinical use. Crit. Rev. Clin. Lab. Sci..

[32] Schosinsky K.H., Lehmann H.P., Beeler M.F. (1974). Measurement of ceruloplasmin from its oxidase activity in serum by use of o-dianisidine dihydrochloride. Clin. Chem..

[33] Siotto M., Pasqualetti P., Marano M., Squitti R. (2014). Automation of *o*-dianisidine assay for ceruloplasmin activity analyses: Usefulness of investigation in Wilson’s disease and in hepatic encephalopathy. J. Neural Transm..

[34] Abe A., Yamashita S., Noma A. (1989). Sensitive, direct colorimetric assay for copper in serum. Clin. Chem..

[35] Squitti R., Ghidoni R., Simonelli I., Ivanova I.D., Colabufo N.A., Zuin M., Benussi L., Binetti G., Cassetta E., Rongioletti M. (2018). Copper dyshomeostasis in Wilson disease and Alzheimer’s disease as shown by serum and urine copper indicators. J. Trace Elem. Med. Biol..

[36] Walshe J.M.B. (2003). Clinical Investigations Standing Committee of the Association of Clinical, Wilson’s disease: The importance of measuring serum caeruloplasmin non-immunologically. Ann. Clin. Biochem..

[37] Twomey P.J., Viljoen A., House I.M., Reynolds T.M., Wierzbicki A.S. (2007). Copper: Caeruloplasmin ratio. J. Clin. Pathol..

[38] Ward N.I., Mason J.A. (1987). Neutron activation analysis techniques for identifying elemental status in Alzheimer’s disease. J. Radioanal. Nucl. Chem..

[39] McIntosh K.G., Cusack M.J., Vershinin A., Chen Z.W., Zimmerman E.A., Molho E.S., Celmins D., Parsons P.J. (2012). Evaluation of a prototype point-of-care instrument based on monochromatic X-ray fluorescence spectrometry: Potential for monitoring trace element status of subjects with neurodegenerative disease. J. Toxicol. Environ. Health A.

[40] Mueller C., Schrag M., Crofton A., Stolte J., Muckenthaler M.U., Magaki S., Kirsch W. (2012). Altered Serum Iron and Copper Homeostasis Predicts Cognitive Decline in Mild Cognitive Impairment. J. Alzheimer’s Dis..

[41] Alimonti A., Ristori G., Giubilei F., Stazi M.A., Pino A., Visconti A., Brescianini S., Monti M.S., Forte G., Stanzione P. (2007). Serum chemical elements and oxidative status in Alzheimer’s disease, Parkinson disease and multiple sclerosis. Neurotoxicology.

[42] Bocca B., Forte G., Petrucci F., Pino A., Marchione F., Bomboi G., Senofonte O., Giubilei F., Alimonti A. (2005). Monitoring of chemical elements and oxidative damage in patients affected by Alzheimer’s disease. Ann. Ist. Super Sanita.

[43] Squitti R., Siotto M., Cassetta E., Idrissi I.G., Colabufo N.A. (2017). Measurements of serum non-ceruloplasmin copper by a direct fluorescent method specific to Cu(II). Clin. Chem. Lab. Med..

[44] Siotto M., Simonelli I., Pasqualetti P., Mariani S., Caprara D., Bucossi S., Ventriglia M., Molinario R., Antenucci M., Rongioletti M. (2016). Association Between Serum Ceruloplasmin Specific Activity and Risk of Alzheimer’s Disease. J. Alzheimer’s Dis..

[45] Zappasodi F., Salustri C., Babiloni C., Cassetta E., del Percio C., Ercolani M., Rossini P.M., Squitti R. (2008). An observational study on the influence of the APOE-epsilon4 allele on the correlation between ‘free’ copper toxicosis and EEG activity in Alzheimer disease. Brain Res..

[46] Squitti R., Lupoi D., Pasqualetti P., Forno G.D., Vernieri F., Chiovenda P., Rossi L., Cortesi M., Cassetta E., Rossini P.M. (2002). Elevation of serum copper levels in Alzheimer’s disease. Neurology.

[47] Plantin L.O., Lying-Tunell U., Kristensson K. (1987). Trace elements in the human central nervous system studied with neutron activation analysis. Biol. Trace Elem. Res..

[48] Corrigan F.M., Reynolds G.P., Ward N.I. (1993). Hippocampal tin, aluminum and zinc in Alzheimer’s disease. Biometals.

[49] Loeffler D.A., Le Witt P.A., Juneau P.L., Sima A.A., Nguyen H.U., De Maggio A.J., Brickman C.M., Brewer G.J., Dick R.D., Troyer M.D. (1996). Increased regional brain concentrations of ceruloplasmin in neurodegenerative disorders. Brain Res..

[50] Deibel M.A., Ehmann W.D., Markesbery W.R. (1996). Copper, iron, and zinc imbalances in severely degenerated brain regions in Alzheimer’s disease: Possible relation to oxidative stress. J. Neurol. Sci..

[51] Squitti R., Quattrocchi C.C., Forno G.D., Antuono P., Wekstein D.R., Capo C.R., Salustri C., Rossini P.M. (2007). Ceruloplasmin (2-D PAGE) Pattern and Copper Content in Serum and Brain of Alzheimer Disease Patients. Biomark Insights.

[52] Religa D., Strozyk D., Cherny R.A., Volitakis I., Haroutunian V., Winblad B., Naslund J., Bush A.I. (2006). Elevated cortical zinc in Alzheimer disease. Neurology.

[53] Magaki S., Raghavan R., Mueller C., Oberg K.C., Vinters H.V., Kirsch W.M. (2007). Iron, copper, and iron regulatory protein 2 in Alzheimer’s disease and related dementias. Neurosci. Lett..

[54] Akatsu H., Hori A., Yamamoto T., Yoshida M., Mimuro M., Hashizume Y., Tooyama I., Yezdimer E.M. (2012). Transition metal abnormalities in progressive dementias. Biometals.

[55] Graham S.F., Nasaruddin M.B., Carey M., Holscher C., McGuinness B., Kehoe P.G., Love S., Passmore P., Elliott C.T., Meharg A.A. (2014). Age-associated changes of brain copper, iron, and zinc in Alzheimer’s disease and dementia with Lewy bodies. J. Alzheimer’s Dis..

[56] Szabo S.T., Harry G.J., Hayden K.M., Szabo D.T., Birnbaum L. (2016). Comparison of Metal Levels between Postmortem Brain and Ventricular Fluid in Alzheimer’s Disease and Nondemented Elderly Controls. Toxicol. Sci..

[57] Xu J., Church S.J., Patassini S., Begley P., Waldvogel H.J., Curtis M.A., Faull R.L.M., Unwin R.D., Cooper G.J.S. (2017). Evidence for widespread, severe brain copper deficiency in Alzheimer’s dementia. Metallomics.

[58] Scholefield M., Church S.J., Xu J., Kassab S., Gardiner N.J., Roncaroli F., Hooper N.M., Unwin R.D., Cooper G.J.S. (2020). Evidence that levels of nine essential metals in post-mortem human-Alzheimer’s-brain and ex vivo rat-brain tissues are unaffected by differences in post-mortem delay, age, disease staging, and brain bank location. Metallomics.

[59] Andrasi E., Farkas E., Scheibler H., Reffy A., Bezur L. (1995). Al, Zn, Cu, Mn and Fe levels in brain in Alzheimer’s disease. Arch. Gerontol. Geriatr..

[60] Lovell M.A., Robertson J.D., Teesdale W.J., Campbell J.L., Markesbery W.R. (1998). Copper, iron and zinc in Alzheimer’s disease senile plaques. J. Neurol. Sci..

[61] Rao K., Rao R., Shanmugavelu P., Menon R. (1999). Trace elements in Alzheimer’s disease: A new hypothesis. Alzheimer’s Rep..

[62] Tandon L., Ni B.F., Ding X.X., Ehmann W.D., Kasarskis E.J., Markesbery W.R. (1994). RNAA for arsenic, cadmium, copper and molybdenum in CNS tissue from subjects with age-related neurodegenerative diseases. J. Radioanal. Nucl. Res..

[63] House E., Esiri M., Forster G., Ince P.G., Exley C. (2012). Aluminium, iron and copper in human brain tissues donated to the Medical Research Council’s Cognitive Function and Ageing Study. Metallomics.

[64] Yoshimasu F., Yasui M., Yase Y., Iwata S., Gajdusek D.C., Gibbs C.J., Chen J.K.M. (1980). Studies on amyotrophic lateral sclerosis by neutron activation analysis-2. Comparative study of analytical results on Guam PD, Japanese ALS and Alzheimer disease cases. Folia Psychiatr. Neurol. Jpn..

[65] Gonzalez C., Martin T., Cacho J., Brenas M.T., Arroyo T., Garcia-Berrocal B., Navajo J.A., Gonzalez-Buitrago J.M. (1999). Serum zinc, copper, insulin and lipids in Alzheimer’s disease epsilon 4 apolipoprotein E allele carriers. Eur. J. Clin. Investig..

[66] Smorgon C., Mari E., Atti A.R., Nora E.D., Zamboni P.F., Calzoni F., Passaro A., Fellin R. (2004). Trace elements and cognitive impairment: An elderly cohort study. Arch. Gerontol. Geriatr..

[67] Sevym S., Ozgur U., Tamer L., Okan D., Aynur O. (2007). Can serum levels of copper and zinc distinguish Alzheimer’s patients from normal subjects?. J. Neurol. Sci..

[68] Agarwal R., Kushwaha S.S., Tripathi C.B., Singh N., Chhillar N. (2008). Serum copper in Alzheimer’s disease and vascular dementia. Indian J. Clin. Biochem..

[69] Azhdarzadeh M., Noroozian M., Aghaverdi H., Akbari S.M., Baum L., Mahmoudi M. (2013). Serum multivalent cationic pattern: Speculation on the efficient approach for detection of Alzheimer’s disease. Sci. Rep..

[70] Lopez N., Tormo C., de Blas I., Llinares I., Alom J. (2013). Oxidative stress in Alzheimer’s disease and mild cognitive impairment with high sensitivity and specificity. J. Alzheimer’s Dis..

[71] Singh N.K., Banerjee B.D., Bala K., Basu M., Chhillar N. (2014). Polymorphism in Cytochrome P450 2D6, Glutathione S-Transferases Pi 1 Genes, and Organochlorine Pesticides in Alzheimer Disease: A Case-Control Study in North Indian Population. J. Geriatr. Psychiatry Neurol..

[72] Park J.H., Lee D.W., Park K.S. (2014). Elevated serum copper and ceruloplasmin levels in Alzheimer’s disease. Asia Pac. Psychiatry.

[73] Paglia G., Miedico O., Cristofano A., Vitale M., Angiolillo A., Chiaravalle A.E., Corso G., di Costanzo A. (2016). Distinctive Pattern of Serum Elements During the Progression of Alzheimer’s Disease. Sci. Rep..

[74] Wang Z.X., Tan L., Wang H.F., Ma J., Liu J., Tan M.S., Sun J.H., Zhu X.C., Jiang T., Yu J.T. (2015). Serum Iron, Zinc, and Copper Levels in Patients with Alzheimer’s Disease: A Replication Study and Meta-Analyses. J. Alzheimer’s Dis..

[75] Talwar P., Grover S., Sinha J., Chandna P., Agarwal R., Kushwaha S., Kukreti R. (2017). Multifactorial Analysis of a Biomarker Pool for Alzheimer Disease Risk in a North Indian Population. Dement. Geriatr. Cogn. Disord..

[76] Rozzini L., Lanfranchi F., Pilotto A., Catalani S., Gilberti M.E., Paganelli M., Apostoli P., Padovani A. (2018). Serum Non-Ceruloplasmin Non-Albumin Copper Elevation in Mild Cognitive Impairment and Dementia due to Alzheimer’s Disease: A Case Control Study. J. Alzheimer’s Dis..

[77] Rembach A., Doecke J.D., Roberts B.R., Watt A.D., Faux N.G., Volitakis I., Pertile K.K., Rumble R.L., Trounson B.O., Fowler C.J. (2013). Longitudinal analysis of serum copper and ceruloplasmin in Alzheimer’s disease. J. Alzheimer’s Dis..

[78] Shere S., Subramanian S., Bharath S., Purushottam M. (2018). Lower levels of serum copper in patients with Alzheimer’s dementia: A controlled study from India. Asian J. Psychiatr..

[79] Basun H., Forssell L.G., Wetterberg L., Winblad B. (1991). Metals and trace elements in plasma and cerebrospinal fluid in normal aging and Alzheimer’s disease. J. Neural Transm. Park Dis. Dement. Sect..

[80] Mattiello G., Gerotto M., Favarato M., Lazzri F., Gasparoni G., Gomirato L., Mazzolini G., Scarpa G., Zanaboni V., Pilone M.G. (1993). Plasma Microelement Analysis from Alzheimer’s and Multi-infartual Dementia Patients. Alzheimer’s Dis. Adv. Clin. Basic Res..

[81] Ashraf A., Stosnach H., Parkes H.G., Hye A., Powell J., So P.-W., AddNeuroMed Consortium (2019). Pattern of Altered Plasma Elemental Phosphorus, Calcium, Zinc, and Iron in Alzheimer’s Disease. Sci. Rep..

[82] Arnal N., Cristalli D.O., de Alaniz M.J., Marra C.A. (2010). Clinical utility of copper, ceruloplasmin, and metallothionein plasma determinations in human neurodegenerative patients and their first-degree relatives. Brain Res..

[83] Alsadany M.A., Shehata H.H., Mohamad M.I., Mahfouz R.G. (2013). Histone deacetylases enzyme, copper, and IL-8 levels in patients with Alzheimer’s disease. Am. J. Alzheimer’s Dis. Other Demen..

[84] Xu J., Church S.J., Patassini S., Begley P., Kellett K.A.B., Vardy E., Unwin R.D., Hooper N.M., Cooper G.J.S. (2018). Plasma metals as potential biomarkers in dementia: A case-control study in patients with sporadic Alzheimer’s disease. Biometals.

[85] Giacconi R., Giuli C., Casoli T., Balietti M., Costarelli L., Provinciali M., Basso A., Piacenza F., Postacchini D., Galeazzi R. (2019). Acetylcholinesterase inhibitors in Alzheimer’s disease influence Zinc and Copper homeostasis. J. Trace Elem. Med. Biol..

[86] Vural H., Demirin H., Kara Y., Eren I., Delibas N. (2010). Alterations of plasma magnesium, copper, zinc, iron and selenium concentrations and some related erythrocyte antioxidant enzyme activities in patients with Alzheimer’s disease. J. Trace Elem. Med. Biol..

[87] Gerhardsson L., Lundh T., Minthon L., Londos E. (2008). Metal concentrations in plasma and cerebrospinal fluid in patients with Alzheimer’s disease. Dement. Geriatr. Cogn. Disord..

[88] Roberts E.A., Schilsky M.L. (2008). American Association for Study of Liver Diseases. Diagnosis and treatment of Wilson disease: An update. Hepatology.

[89] European Association for Study of the Liver (2012). EASL Clinical Practice Guidelines: Wilson’s disease. J. Hepatol..

[90] Reed E., Lutsenko S., Bandmann O. (2018). Animal models of Wilson disease. J. Neurochem..

[91] Fujiwara N., Iso H., Kitanaka N., Kitanaka J., Eguchi H., Ookawara T., Ozawa K., Shimoda S., Yoshihara D., Takemura M. (2006). Effects of copper metabolism on neurological functions in Wistar and Wilson’s disease model rats. Biochem. Biophys. Res. Commun..

[92] Squitti R., Ventriglia M., Gennarelli M., Colabufo N.A., el Idrissi I.G., Bucossi S., Mariani S., Rongioletti M., Zanetti O., Congiu C. (2017). Non-Ceruloplasmin Copper Distincts Subtypes in Alzheimer’s Disease: A Genetic Study of ATP7B Frequency. Mol. Neurobiol..

[93] Squitti R., Ghidoni R., Siotto M., Ventriglia M., Benussi L., Paterlini A., Magri M., Binetti G., Cassetta E., Caprara D. (2014). Value of serum nonceruloplasmin copper for prediction of mild cognitive impairment conversion to Alzheimer disease. Ann. Neurol..

[94] Squitti R., Pasqualetti P., Polimanti R., Salustri C., Moffa F., Cassetta E., Lupoi D., Ventriglia M., Cortesi M., Siotto M. (2013). Metal-score as a potential non-invasive diagnostic test for Alzheimer’s disease. Curr. Alzheimer Res..

[95] Squitti R., Bressi F., Pasqualetti P., Bonomini C., Ghidoni R., Binetti G., Cassetta E., Moffa F., Ventriglia M., Vernieri F. (2009). Longitudinal prognostic value of serum “free” copper in patients with Alzheimer disease. Neurology.

[96] Singh I., Sagare A.P., Coma M., Perlmutter D., Gelein R., Bell R.D., Deane R.J., Zhong E., Parisi M., Ciszewski J. (2013). Low levels of copper disrupt brain amyloid-beta homeostasis by altering its production and clearance. Proc. Natl. Acad. Sci. USA.

[97] Coelho F.C., Squitti R., Ventriglia M., Cerchiaro G., Daher J.P., Rocha J.G., Rongioletti M.C.A., Moonen A.C. (2020). Agricultural Use of Copper and Its Link to Alzheimer’s Disease. Biomolecules.

[98] Voss K., Harris C., Ralle M., Duffy M., Murchison C., Quinn J.F. (2014). Modulation of tau phosphorylation by environmental copper. Transl. Neurodegener..

[99] National Research Council (2020). Copper in Drinking Water.

[100] Barnard N.D., Bush A.I., Ceccarelli A., Cooper J., de Jager C.A., Erickson K.I., Fraser G., Kesler S., Levin S.M., Lucey B. (2014). Dietary and lifestyle guidelines for the prevention of Alzheimer’s disease. Neurobiol. Aging.

[101] Squitti R., Siotto M., Polimanti R. (2014). Low-copper diet as a preventive strategy for Alzheimer’s disease. Neurobiol. Aging.

[102] McKhann G., Drachman D., Folstein M., Katzman R., Price D., Stadlan E.M. (1984). Clinical diagnosis of Alzheimer’s disease: Report of the NINCDS-ADRDA Work Group under the auspices of Department of Health and Human Services Task Force on Alzheimer’s Disease. Neurology.

[103] Diouf I., Bush A.I., Ayton S., Alzheimer’s Disease Neuroimaging Initiative (2020). Cerebrospinal fluid ceruloplasmin levels predict cognitive decline and brain atrophy in people with underlying beta-amyloid pathology. Neurobiol. Dis..

