# Supplementary material for: Copper Imbalance in Alzheimer’s Disease: Meta-Analysis of Serum, Plasma, and Brain Specimens, and Replication Study Evaluating ATP7B Gene Variants
Source: Biomolecules. 2021 Jun 29;11(7):960. doi: 10.3390/biom11070960 (PMC8301962; doi:10.3390/biom11070960)
Supplement: Supplementary file 1 [file biomolecules-11-00960-s001.zip › biomolecules-1228899-supplementary.pdf]

biomolecules

MDPI

Supplementary Materials

Copper Imbalance in Alzheimer's Disease: Meta-Analysis of Serum, Plasma, and Brain Specimens, and Replication Study Evaluating *ATP7B* Gene Variants

Rosanna Squitti [1,*], Mariacarla Ventriglia [2], Ilaria Simonelli [2], Cristian Bonvicini [1], Alfredo Costa [3,4], Giulia Perini [3,4], Giuliano Binetti [5], Luisa Benussi [1], Roberta Ghidoni [1], Giacomo Koch [6,7], Barbara Borroni [8], Alberto Albanese [9], Stefano L. Sensi [10,11,12,*] and Mauro Rongioletti [13]

1 Molecular Markers Laboratory, IRCCS Istituto Centro San Giovanni di Dio Fatebenefratelli, 25125 Brescia, Italy; cbonvicini@fatebenefratelli.eu (C.B.); lbenussi@fatebenefratelli.eu (L.B.); rghidoni@fatebenefratelli.eu (R.G.)
2 Fatebenefratelli Foundation for Health Research and Education, AFaR Division, San Giovanni Calibita Fatebenefratelli Hospital, Isola Tiberina, 00186 Rome, Italy; mariacarla.ventriglia@afar.it (M.V.); ilaria.simonelli@afar.it (I.S.)
3 Unit of Behavioral Neurology, IRCCS Mondino Foundation, 27100 Pavia, Italy; alfredo.costa@unipv.it (A.C.); giulia.perini01@universitadipavia.it (G.P.)
4 Department of Brain and Behavior, University of Pavia, 27100 Pavia, Italy
5 MAC Memory Clinic and Molecular Markers Laboratory, IRCCS Istituto Centro San Giovanni di Dio Fatebenefratelli, 25125 Brescia, Italy; gbinetti@fatebenefratelli.eu
6 Section of Human Physiology, University of Ferrara, 44121 Ferrara, Italy; g.koch@hsantalucia.it
7 Department of Clinical and Behavioural Neurology, IRCCS Santa Lucia Foundation, 00179 Rome, Italy
8 Centre for Neurodegenerative Disorders, Department of Clinical and Experimental Sciences, University of Brescia, 25123 Brescia, Italy; barbara.borroni@unibs.it
9 Department of Neurology, IRCCS Istituto Clinico Humanitas, Rozzano, 20089 Milan, Italy; alberto.albanese@humanitas.it
10 Department of Neuroscience, Imaging and Clinical Science, "G. D'Annunzio" University of Chieti-Pescara, 66100 Chieti, Italy
11 Institute for Mind Impairments and Neurological Disorders—iMIND, University of California-Irvine, Irvine, CA 92697, USA
12 Molecular Neurology Units, Center for Advanced Studies and Technology (CAST), University G. D'Annunzio of Chieti-Pescara, 66100 Chieti, Italy
13 Department of Laboratory Medicine, Research and Development Division, San Giovanni Calibita Fatebenefratelli Hospital, Isola Tiberina, 00186 Rome, Italy; maurociroantonio.rongioletti@fbf-isola.it
* Correspondence: rosanna.squitti@afar.it (R.S.); ssensi@uci.edu (S.L.S.)

Table S1. Demographic data of the eligible studies for meta-analysis of copper markers in serum/plasma.

	Alzheimer's dementia				Healthy controls			
References (Authors, year)	N	Sex (%F)	Mean age (SD)	MMSE Mean (SD)/ Median (25-75th percentile)	N	Sex (%F)	Mean age (SD)	MMSE Mean (SD)/ Median (25-75th percentile)
Giacconi et al. 2019 [1]	95	67%	77.5 (0.06)	20.2 (0.04)	84	80%	72.9 (0.07)	28.1 (0.01)
Ashraf et al. 2019 [2]	44	54%	77.5 (6.2)	20.8 (4.6)	44	54%	76 (5.3)	28.9 (1.3)
Shere et al. 2018 [3]	44	54%	68.3 (9.7)	13.7 (6.6)	52	48%	65.7 (7.6)	29.5 (1.1)
Rozzini et al. 2018 [4]	44	61%	77.1 (7.6)	18.1 (5.6)	28	43%	76.1 (8.4)	29.3 (1.0)
Xu et al. 2018 [5]	42	47.6%	78.2 (1.2)	21 (11-25)	43	46.5%	78.1 (1.1)	29 (27-30)
Squitti et al. 2018 [6]	385	73%	76 (2.5)	≤25	336	60%	65 (3.5)	
Squitti et al. 2017 [7]	89	67.5%	73 (8.5)	19 (4.7)	147	52%	49 (12.7)	29 (1)
Talwar et al. 2017 [8]	108	39%	68.8 (9.2)	13.5 (5.7)	159	48%	63.2 (7.5)	28.2 (1.5)
Pu et al. 2017 [9]	28	46%	73.4 (6.0)	19.8 (3.3)	40	47%	74.2 (6.5)	24.2 (3.3)
Pu et al. 2017 [9]	42	45%	75.8 (6.7)	13.5 (3)				
Pu et al. 2017 [9]	55	45%	77.8 (6.5)	5.6 (1.1)				
Siotto et al. 2016 [10]	84	69%	77 (8.7)	<25	58	47%	64.5 (18)	<25
Paglia et al. 2016 [11]	34	74%	72.4 (7.5)	12.3 (8.2)	40	63%	65.5 (6.4)	29.6 (0.7)
Koc et al. 2015 [12]	45	49%	77.7 (9.3)		33	51%	73.2 (10.6)	
Wang et al. 2015 [13]	83	64%	74 (7.1)		83	63%	72.2 (7.5)	
Gonzalez-Dominguez et al. 2014 [14]	30	60%	80.9 (4.5)		30	57%	74 (5.7)	
Singh et al. 2014 [15]	100	39%	62.7 (7.2)		100	39%	59.7 (8.1)	
Park et al. 2014 [16]	89	54%	77.8 (6.6)		118	58%	69.9 (5.9)	
Azhdarzadeh et al. 2013 [17]	30				20			
Azhdarzadeh et al. 2013 [17]	50				50			
Lopez et al. 2013 [18]	36	55%	77.7 (5.3)	20.7 (4.4)	33	64%	74 (5.0)	28.9 (1.3)
Rembach et al. 2013 [19]	152	59%	77 (7.9)	18.9 (5.3)	716	58%	69 (6.8)	28.9 (1.2)
Alsadany et al. 2012 [20]	25	56%	72.2 (5.9)	12.6 (5.8)	25	52%	72.8 (4.1)	27.8 (4.4)
Brewer et al. 2010 [21]	28	46%	76.2	24 (3.9)	29	69%	68.6	29.8 (0.7)
Baum et al. 2010 [22]	44	66%	74.3 (8.7)	<30	41	49%	79.1 (6.0)	<30

Arnal et al. 2010 [23]	110	56%	70 (5.6)	22.2 (3.1)	79	48%	77.8 (3.7)	27.8 (1.1)
Vural et al. 2010 [24]	50	54%	72 (6.8)		50	52%	65.1 (7.1)	
Agarwal et al. 2008 [25]	50	38%	59.9 (11.6)	14.1 (7.6)	50	34%	55.3 (10.9)	
Zappasodi et al. 2008 [26]	54	81%	73.7 (8.7)	19.5 (3.8)	20	65%	71.5 (9.2)	28 (1.7)
Gherardsson et al. 2008 [27]	173	70.5%	75 (52-86)	22 (2-30)	54	66.7%	73 (60-94)	30 (28-30)
Sevym et al. 2007 [28]	98	66%	72.1 (6.7)		76	59%	70.3 (5.7)	
Sedighi et al. 2006 [29]	50	48%	76.4	14.3 (4.6)	50	50%	67.8	25.8 (1.5)
Bocca et al. 2005 [30]	60	67%	74.6 (6.4)	2-28	44	75%	>45	
Smorgon et al. 2004 [31]	8		79 (5)		11		78 (9.0)	
Ozcakaya and Delibas 2002 [32]	27	29%	72.3 (6.5)	16.8 (1.3)	25	36%	64.4 (7.2)	28.2 (2.4)
Squitti et al. 2002 [33]	79	68%	74.5 (7.4)	17.3 (4.9)	76	57%	70.1 (10.8)	27.7 (2.2)
Gonzalez et al. 1999 [34]	51	71%	74.5 (2.3)		40	45%	70.3 (4.0)	
Molina et al. 1998 [35]	26	46%	73.1 (8.2)	13.2 (5.7)	28	43%	70.8 (7.3)	
Snaedel et al. 1998 [36]	44	73%	74.3 (53-89)	15.9 (6-28)	44	matched	matched	
Molaschi et al. 1996 [37]	31	100%	77.2 (2.4)		421	100%	77.6 (2.3)	
Mattiello et al. 1993 [38]	21	71%	81 (6)		10	50%	82 (2.0)	
Basun et al. 1991 [39]	24	71%	75 (8)		28	57%	78 (3.0)	
Jeandel et al. 1989 [40]	55	73%	81.7 (5.7)	<25	24	40%		
Kapaki et al. 1989 [41]	5	20%	54		28	36%	46	
Shore et al. 1984 [42]	10	30%	63.7 (8.4)		10	70%	61.9 (8.0)	
Present study	97	64%	70.4 (7.0)	17.9 (5.8)	70	0.77	67.1 (9.2)	28.3 (1.9)

Table S2. Serum or plasma Cu levels in Alzheimer's dementia patients and in healthy controls.

		Alzhimer's dementia		Healthy controls	
Study	Sample	N	Cu, μmol/L (SD)	N	Cu, μmol/L (SD)
Giacconi et al. 2019 [1]	plasma	95	17.2 (0.03)	84	17.1 (0.03)
Ashraf et al. 2019 [2]	plasma	44	16.1 (6.9)	44	17 (6.6)
Rozzini et al. 2018 [4]	serum	44	18.3 (3.5)	28	16.0 (3.3)
Squitti et al. 2018 [6]	serum	385	15.2 (1)	336	13.2 (0.9)
Shere et al. 2018 [3]	serum	44	16.1 (3.7)	52	18.2 (4.4)
Xu et al. 2018 [5]	plasma	42	14.4 (1.4)	43	13.5 (1.3)
Squitti et al 2017 [7]	serum	89	15.9 (3.6)	147	16.0 (6.4)
Talwar et al. 2017 [8]	serum	108	22.1 (9.6)	159	18.7 (7.0)
		28	16.3 (6.4)		
Pu et al. 2017 [9] *	serum	42	20.3 (6.7)	40	16.3 (6.5)
		55	21.3 (6.9)		
Siotto et al. 2016 [10]	serum	84	15.5 (3.3)	58	14.0 (2.3)
Paglia et al. 2016 [11]	serum	34	12.8 (3.2)	40	11.1 (3.8)
Koc et al. 2015 [12]	serum	45	14.2 (10.5)	33	15.9 (11.7)
Wang et al. 2015 [13]	serum	83	19.0 (3.6)	83	16.1 (2.8)
Gonzalez-Dominguez et al. 2014 [14]	serum	30	17.5 (4.2)	30	16.5 (3.2)
Singh et al. 2014 [15]	serum	100	18.3 (0.5)	100	14.9 (0.3)
Park et al. 2014 [16]	serum	89	18.0 (3.0)	118	17.0 (3.2)
Azhdarzadeh et al. 2013 [17]	serum	30	17.0 (3.1)	20	15.6 (2.2)
Azhdarzadeh et al. 2013 [17]	serum	50	15.8 (1.5)	50	15.1 (1.9)
Lopez et al. 2013 [18]	serum	36	15.8 (2.9)	33	13.8 (3.7)
Rembach et al. 2013 [19]	serum	152	13.9 (2.7)	716	14.6 (2.9)
Alsadany et al. 2012 [20]	plasma	25	17.3 (1.9)	25	12.3 (2)
Brewer et al. 2010 [21]	serum	28	17.0 (2.4)	29	18.4 (3.1)
Baum et al. 2010 [22]	serum	44	16.2 (3.5)	41	15.3 (2.7)
Arnal et al. 2010 [23]	plasma	110	15.7 (0.6)	79	12.6 (0.8)
Vural et al. 2010 [24]	plasma	50	20.7 (2.9)	50	22.5 (2.8)
Agarwal et al. 2008 [25]	serum	50	24.6 (4.8)	50	21.2 (5.0)
Zappasodi et al. 2008 [26]	serum	54	15.1 (3.4)	20	12.9 (3.0)
Gherardsson et al. 2008 [27]	plasma	173	22 (16.4)	54	28.4 (30.2)
Sevym et al. 2007 [28]	serum	98	16.7 (2.9)	76	15.4 (2.1)
Sedighi et al. 2006 [29]	serum	50	21.7 (3.1)	50	20.9 (2.5)
Bocca et al. 2005 [30]	serum	60	15.2 (3.9)	44	14.3 (3.1)
Smorgon et al. 2004 [31]	serum	8	22.9 (3.9)	11	16.7 (1.3)
Ozcakaya and Delibas 2002 [32]	serum	27	12.0 (0.2)	25	12.1 (0.2)
Squitti et al. 2002 [33]	serum	79	18.3 (5.7)	76	13.7 (2.6)
Gonzalez et al. 1999 [34]	serum	51	16.6 (1.5)	40	15.4 (1.2)
Molina et al. 1998 [35]	serum	26	15.1 (3.5)	28	14.5 (4.1)
Snaedel et al. 1998 [36]	serum	44	19.1 (3.9)	44	19.4 (2.2)
Molaschi et al. 1996 [37]	serum	31	18.9 (3.4)	421	19.3 (3.8)
Mattiello et al. 1993 [38]	plasma	21	18.8 (0.2)	10	16.7 (0.3)
Basun et al. 1991 [39]	plasma	24	17.0 (3.9)	28	16.7 (3.0)
Jeandel et al. 1989 [40]	serum	55	22.0 (6.1)	24	21.2 (4.1)
Kapaki et al. 1989 [41] #903}	serum	5	14.2 (3.8)	28	16.2 (2.2)

Shore et al. 1984 [42]	serum	10	18.3 (4.2)	10	18.6 (1.6)
Present study	serum	97	16.3 (4.6)	70	14.8 (3.2)

*Data were pooled for the analyses. SD: standard deviation

Table S3. Indices of serum Cu status in all the 18 studies employed for the meta-analysis of non-Ceruloplasmin Cu (Non-Cp Cu) and ceruloplasmin (Cp).

	Alzheimer's dementia							Healthy controls							
**Study (Authors, year)**	**N**	**Sex (F%)**	**Mean age (SD)**	**MMSE (SD)**	**Cu µmol/L (SD)**	**Cp mg/dl (SD)**	**Non-Cp Cu µmol/L (SD)**	**N**	**Female (%)**	**Mean age (SD)**	**MMSE (SD)**	**Cu µmol/L (SD)**	**Cp mg/dl (SD)**	**Non-Cp Cu µmol/L (SD)**	**Cu:Cp ratio**
Rozzini et al, 2018 [4]	44	61%	77.1 (7.6)	18.1 (5.6)	18.3 (3.5)	-	0.57 (0.34)	28	43%	76.1 (8.4)	29.3 (1.0)	16 (3.3)	-	0.3 (0.11)	n.a.
Squitti et al, 2018 [6]	385	73%	76 (2.5)	≤25	15.2 (13-17)*	27.4 (5.2)	2.2 (2.1)	336	60%	65 (3.5)		13.2 (11.3-15)*	27.1 (5.0)	0.4 (2.1)	6.5
Shere et al, 2018 [3]	44	54%	68.3 (9.7)	13.7 (6.6)	16.1 (3.7)	22.6 (4.9)	5.4 (1.8)	52	48%	65.7 (7.6)	29.5 (1.1)	18.2 (4.4)	23.3 (4.7)	7.2 (2.3)	n.a.
Squitti et al, 2017 [7]	89	67.5%	73 (8.5)	16 (4.7)	15.9 (3.59)	28.8 (5.59)	2.31 (1.64)	147	52.7%	49 (12.7)	29 (1)	16.04 (6.39)	30.48 (10.38)	1.68 (2.46)	6.8
Talwar et al, 2017 [8]	108	39%	68.8 (9.2)	13.5 (5.7)	22.1 (9.6)	36.1 (9.3)	5.0 (5.2)	159	48%	63.2 (7.5)	28.2 (1.5)	18.7 (7.0)	36.6 (10.8)	1.4 (1.9)	6.7
	284	46%	73.4 (6.0)	19.8 (3.3)	16.9 (6.4)	22 (52)	6.5 (22.1)								
Pu et al, 2017 [9] **	42	45%	75.8 (6.7)	13.5 (3.0)	20.3 (6.7)	17 (48)	12.1 (20.1)	40	47%	74.2 (6.5)	24.2 (3.3)	16.3 (6.5)	24 (54)	27.6	8.96
	55	45%	77.8 (6.5)	5.62 (1.1)	21.3 (6.9)	15 (41)	13.5 (17.0)								
Siotto et al, 2016 [10]	84	69%	77 (8.7)	≤25	15.5 (3.3)	28.2 (4.3)	2.1 (1.8)	58	47%	64.5 (18)		14 (2.3)	26.3 (3.7)	1.5 (1.5)	7
Park et al, 2014 [16]	64	50%	75.0 (5.4)	17.9 (5.1)	28.4 (4.7)	23.3 (4.1)	7.0 (1.9)	67	55%	73.4 (5.3)	24.2 (3.8)	26.7 (5.0)	21.7 (4.9)	6.7 (2.2)	10.3
Lopez et al, 2013 [18]	36	55%	77.7 (5.3)	20.7 (4.4)	15.8 (2.9)	25.9 (4.1)	3.6 (2.1)	33	64%	74.0 (5.0)	28.9 (1.3)	13.8 (3.7)	25.8 (4.3)	1.6 (3.0)	7.06
Rembach et al, 2013 [19]	152	59%	77 (7.9)	18.9 (5.3)	13.9 (2.7)	31.6 (7.9)	-0.6 (2.7)	716	58%	69.0 (6.8)	28.9 (1.2)	14.6 (2.9)	31.7 (7.9)	-0.6 (3.1)	5.97
Brewer et al, 2010 [21]	28	46%	76.2	24 (3.9)	17 (2.4)	24.4 (3.0)	5.5 (1.8)	29	69%	68.6	29.8 (0.7)	18.4 (3.1)	25.7 (3.7)	6.3 (2.5)	9.45

Arnal et al, 2010 [23]	110	56%	70 (5.6)	22.2 (3.1)	15.6 (3.1)	25.2 (10.7)	3.8 (4.5)	79	48%	77.8 (3.7)	27.8 (1.1)	12.7 (0.2)	26.5 (7.0)	0.2 (2.8)	6.34
Agarwal et al, 2008 [25]	50	38%	60.0 (11.6)	14.1 (7.6)	24.6 (4.8)	41.6 (9.6)	4.9 (3.7)	50	34%	55.3 (10.9)		21.2 (5.0)	28.8 (8.0)	7.5 (4.1)	9.68
Zappasodi et al, 2008 [26]	54	81%	73.7 (8.7)	19.5 (3.8)	15.1 (3.4)	27.6 (5.9)	2.1 (2.5)	20	65%	71.5 (9.2)	28	12.9 (3.0)	27 (5.3)	0.2 (2.5)	6.31
Sedighi et al, 2006 [29]	50	48%	76.4	14.3 (4.6)	21.7 (3.1)	27.7 (9.6)	8.6 (3.3)	50	50%	67.8	25.8	20.8 (2.5)	31.1 (5.4)	6.2 (2.2)	8.85
Snaedal et al, 1998 [36]	44	73%	74.3 (53-89)	15.9 (6-28)	19.1 (3.9)	38.2 (6.8)	1.1 (2.9)	44	matched	matched		19.4 (2.2)	38.3 (8.1)	1.3 (3.0)	6.69
Molaschi et al, 1996 [37]	31	100%	77.2 (2.4)		18.9 (3.4)	38.8 (7.1)	0.5 (2.7)	421	100%	77.6 (2.3)		19.3 (3.8)	40.6 (9.1)	0.1 (3.6)	6.27
Present study	97	64%	70.4 (7.0)	17.9 (5.8)	16.3 (4.6)	29.4 (4.7)	2.39 (3.4)	73	77%	67.1 (9.2)	28.3	14.8 (3.2)	29.5 (4.3)	0.86 (1.8)	6.6

*25-75th percentiles. **Data were pooled for the analyses. Abbreviations: F, female; SD, standard deviation; MMSE, minimental examination test.

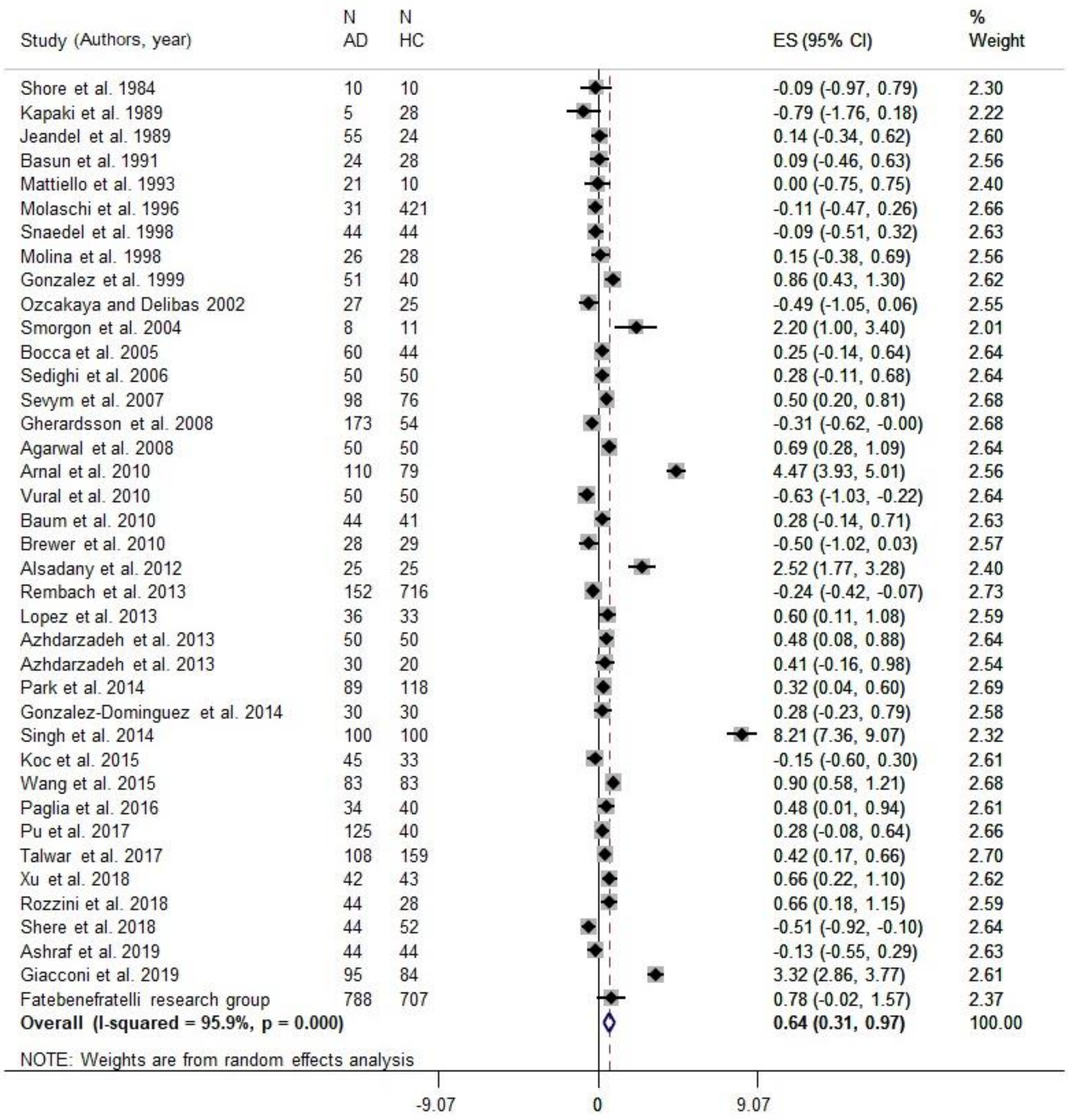

Figure S1. Standardized mean difference (SMD) computed from the studies on Cu serum/plasma levels (μmol/L) in AD patients and HC subjects. SMDs between patients and controls are represented by squares, whose sizes are proportional to the sample size of the relative study. The whiskers represent the 95% confidence interval (CI). The diamond represents the pooled estimate based on the random-effects model, with the centre representing the point estimate and the width indicating the associated 95% CI. All the studies produced by the Fatebenefratelli research group were pooled together and considered as a single study. Abbreviations: PY, publication year; N, number; SD, standard deviation; HC, healthy controls.

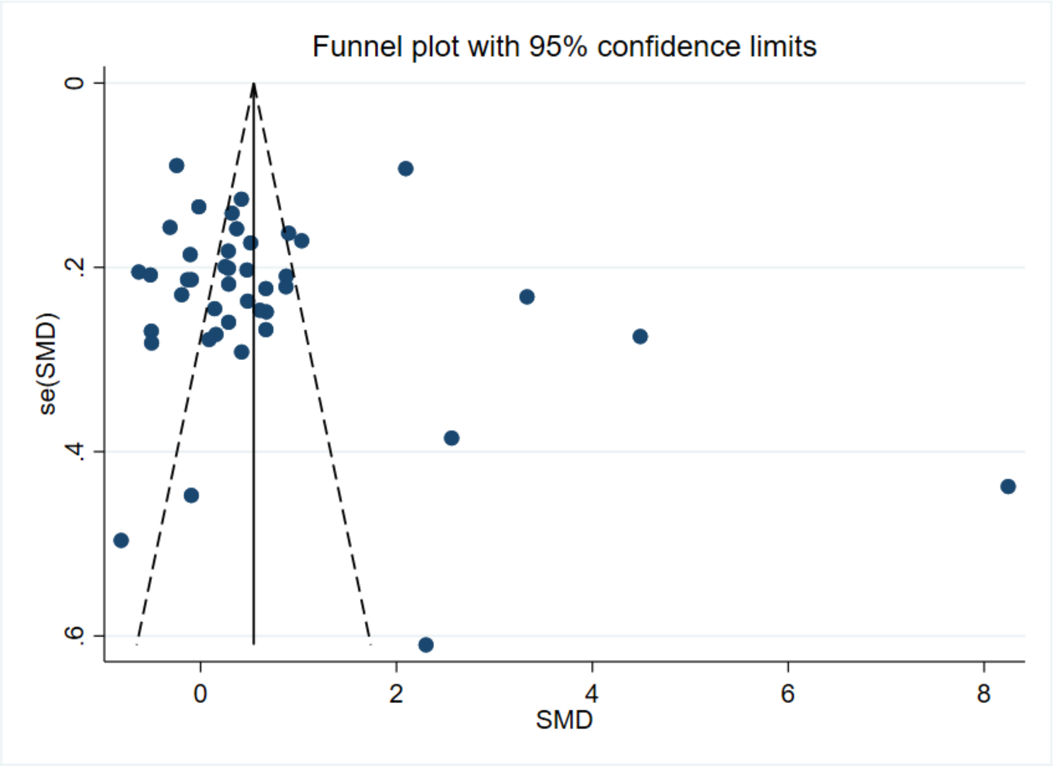

Figure S2. Funnel plot suggested no presence of publication bias in the studies of Cu in serum/plasma evaluated in Alzheimer's dementia and Healthy Controls.

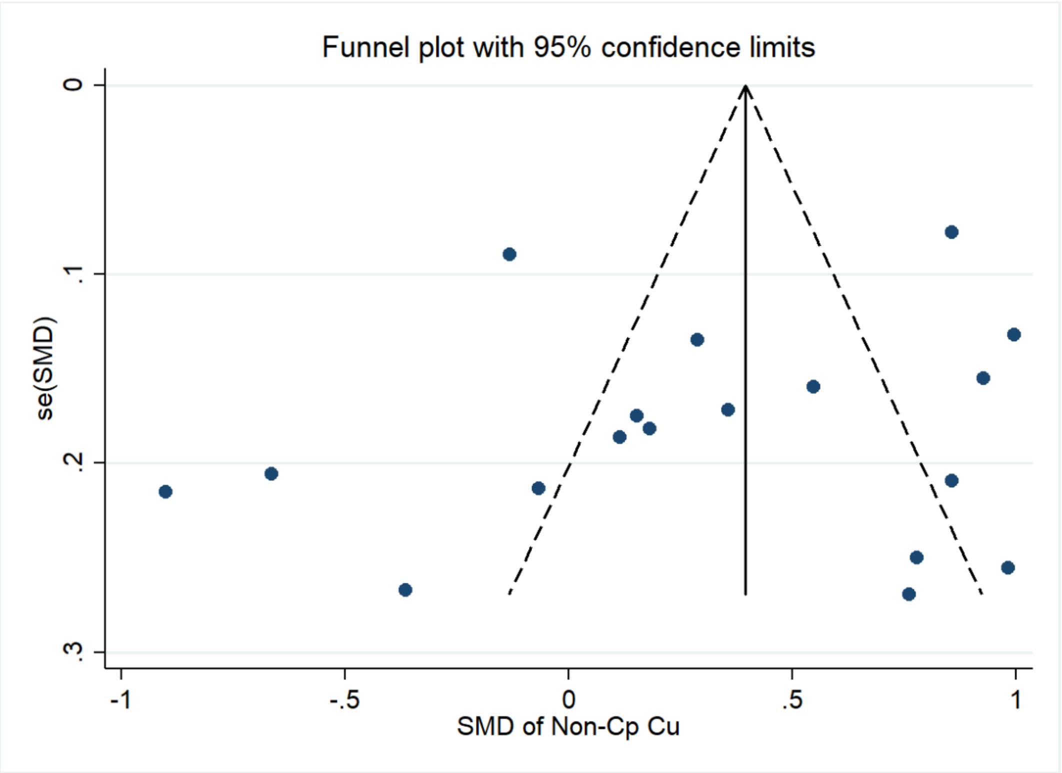

Figure S3. Funnel plot suggested no presence of publication bias in the studies of non-ceruloplasmin Cu in serum/plasma evaluated in Alzheimer's dementia and Healthy Controls.

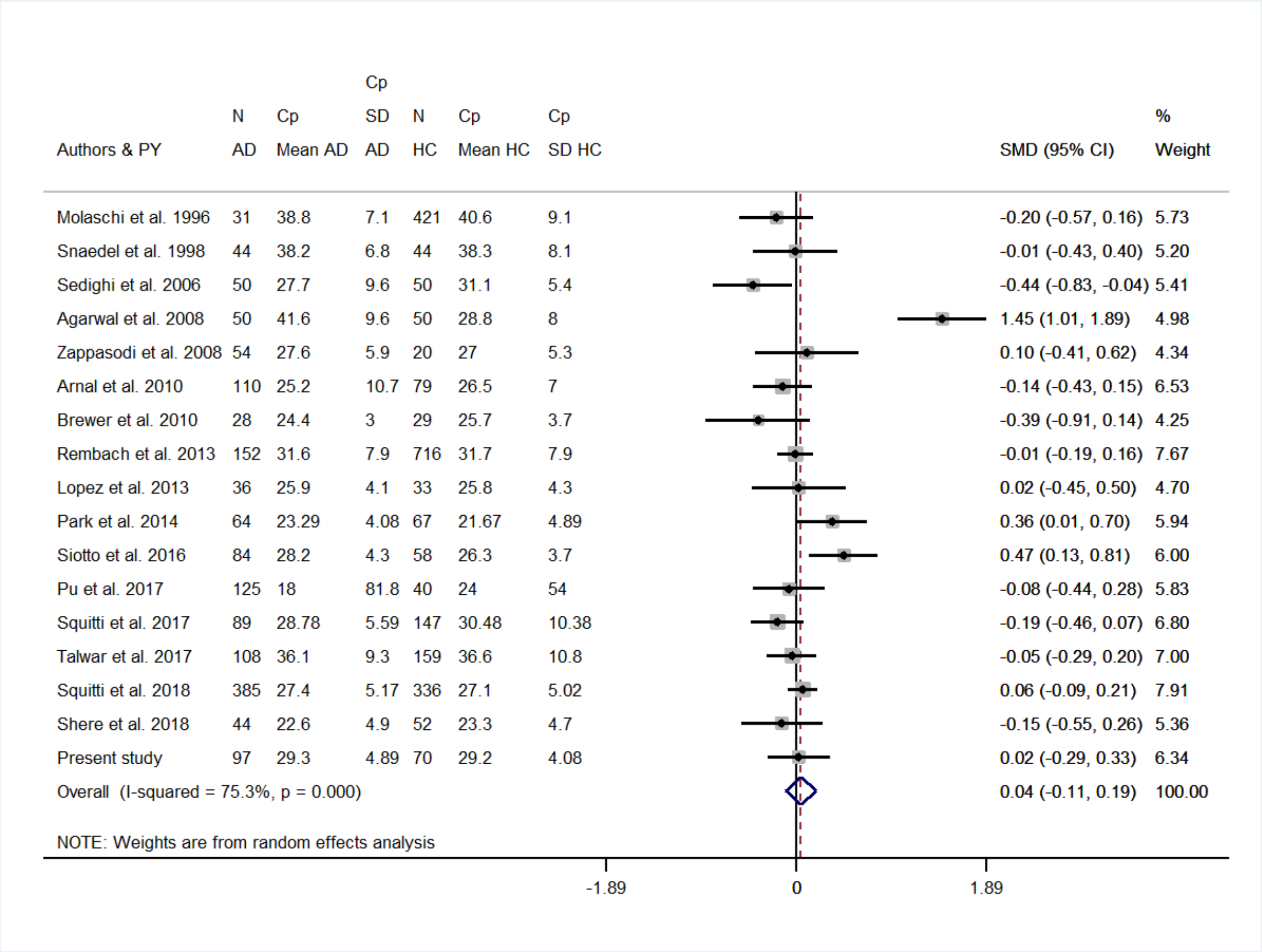

Figure S4. Standardized mean difference (SMD) computed from the studies on ceruloplasmin (mg/dL) in Alzheimer's dementia patients and Healthy Controls.

SMDs between AD subjects and controls are represented by squares, whose sizes are proportional to the sample size of the relative study. The whiskers represent the 95% confidence interval (CI). The diamond represents the pooled estimate based on the random effects model, with the centre representing the point estimate and the width the associated 95% CI.

Most of the ceruloplasmin studies carried out in serum/plasma demonstrated no variation (15 studies), while 2 studies reported increases and a study decreased values of ceruloplasmin in AD patients in comparison with healthy controls. As a whole, the forest plot of the meta-analysis reveals no variation of ceruloplasmin in serum/plasma of AD patients in comparison with healthy controls as indicated by the diamond that touch the zero line. This result further confirm that the increase in serum/plasma Cu can be explained by the increased levels of non-ceruloplasmin Cu fraction, as depicted in previous studies and in the current dedicated meta-analyses.

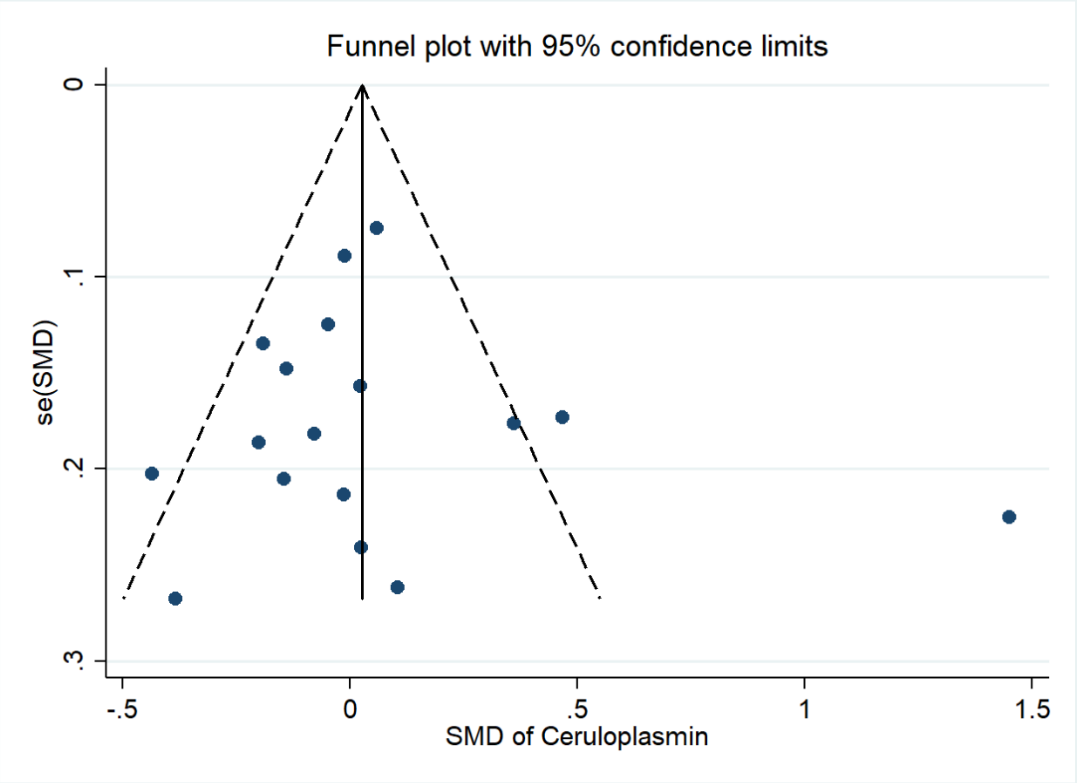

Figure S5. Funnel plot suggested no presence of publication bias in the studies of ceruloplasmin in serum/plasma evaluated in Alzheimer's disease and healthy controls.

References

1. Giacconi, R.; C. Giuli; T. Casoli; M. Balietti; L. Costarelli; M. Provinciali; A. Basso; F. Piacenza; D. Postacchini; R. Galeazzi; P. Fattoretti; L. Nisi; P. Fabbietti; R. PapaM. Malavolta, Acetylcholinesterase inhibitors in Alzheimer's disease influence Zinc and Copper homeostasis. *J Trace Elem Med Biol*, **2019**. 55,58-63.
2. Ashraf, A.; H. Stosnach; H.G. Parkes; A. Hye; J. Powell; P.W. Soc. AddNeuroMed, Pattern of Altered Plasma Elemental Phosphorus, Calcium, Zinc, and Iron in Alzheimer's Disease. *Sci Rep*, **2019**. 9,3147.
3. Shere, S.; S. Subramanian; S. BharathM. Purushottam, Lower levels of serum copper in patients with Alzheimer's dementia: A controlled study from India. *Asian J Psychiatr*, **2018**. 32,73-74.
4. Rozzini, L.; F. Lanfranchi; A. Pilotto; S. Catalani; M.E. Gilberti; M. Paganelli; P. ApostoliA. Padovani, Serum Non-Ceruloplasmin Non-Albumin Copper Elevation in Mild Cognitive Impairment and Dementia due to Alzheimer's Disease: A Case Control Study. *J Alzheimers Dis*, **2018**. 61,907-912.
5. Xu, J.; S.J. Church; S. Patassini; P. Begley; K.A.B. Kellett; E. Vardy; R.D. Unwin; N.M. HooperG.J.S. Cooper, Plasma metals as potential biomarkers in dementia: a case-control study in patients with sporadic Alzheimer's disease. *Biometals*, **2018**. 31,267-276.
6. Squitti, R.; R. Ghidoni; I. Simonelli; I.D. Ivanova; N.A. Colabufo; M. Zuin; L. Benussi; G. Binetti; E. Cassetta; M. RongiolettiM. Siotto, Copper dyshomeostasis in Wilson disease and Alzheimer's disease as shown by serum and urine copper indicators. *J Trace Elem Med Biol*, **2018**. 45,181-188.
7. Squitti, R.; M. Siotto; E. Cassetta; I.G. IdrissiN.A. Colabufo, Measurements of serum non-ceruloplasmin copper by a direct fluorescent method specific to Cu(II). *Clin Chem Lab Med*, **2017**. 55,1360-1367.
8. Talwar, P.; S. Grover; J. Sinha; P. Chandna; R. Agarwal; S. KushwahaR. Kukreti, Multifactorial Analysis of a Biomarker Pool for Alzheimer Disease Risk in a North Indian Population. *Dement Geriatr Cogn Disord*, **2017**, 44,25-34.
9. Pu, Z.; W. Xu; Y. Lin; J. HeM. Huang, Oxidative Stress Markers and Metal Ions are Correlated With Cognitive Function in Alzheimer's Disease. *Am J Alzheimers Dis Other Demen*, **2017**.1533317517709549.10.1177/1533317517709549 DOI|.

10. Siotto, M.; I. Simonelli; P. Pasqualetti; S. Mariani; D. Caprara; S. Bucossi; M. Ventriglia; R. Molinario; M. Antenucci; M. Rongioletti; P.M. RossiniR. Squitti, Association Between Serum Ceruloplasmin Specific Activity and Risk of Alzheimer's Disease. *J Alzheimers Dis* **2016**. 50,1181-1189.
11. Paglia, G.; O. Miedico; A. Cristofano; M. Vitale; A. Angiolillo; A.E. Chiaravalle; G. CorsoA. Di Costanzo, Distinctive Pattern of Serum Elements During the Progression of Alzheimer's Disease. *Sci Rep,* **2016**. 6,22769.
12. Koc, E.R.; A. Ilhan; A. Zubeyde; B. Acar; M. Gurler; A. Altuntas; M. KarapirliA.S. Bodur, A comparison of hair and serum trace elements in patients with Alzheimer disease and healthy participants. *Turk J Med Sci,* **2015**. 45,1034-1039.
13. Wang, Z.X.; L. Tan; H.F. Wang; J. Ma; J. Liu; M.S. Tan; J.H. Sun; X.C. Zhu; T. JiangJ.T. Yu, Serum Iron, Zinc, and Copper Levels in Patients with Alzheimer's Disease: A Replication Study and Meta-Analyses. *J Alzheimers Dis,* **2015**. 47,565-581.
14. Gonzalez-Dominguez, R.; T. Garcia-BarreraJ.L. Gomez-Ariza, Characterization of metal profiles in serum during the progression of Alzheimer's disease. *Metallomics,* **2014**. 6,292-300.
15. Singh, B.; A.K. Parsaik; M.M. Mielke; P.J. Erwin; D.S. Knopman; R.C. PetersenR.O. Roberts, Association of mediterranean diet with mild cognitive impairment and Alzheimer's disease: a systematic review and meta-analysis. *J Alzheimers Dis,* **2014**. 39,271-282.
16. Park, J.H.; D.W. LeeK.S. Park, Elevated serum copper and ceruloplasmin levels in Alzheimer's disease. *Asia Pac Psychiatry,* **2014**. 6,38-45.
17. Azhdarzadeh, M.; M. Noroozian; H. Aghaverdi; S.M. Akbari; L. BaumM. Mahmoudi, Serum multivalent cationic pattern: speculation on the efficient approach for detection of Alzheimer's disease. *Sci Rep,* **2013**. 3,2782.
18. Lopez, N.; C. Tormo; I. De Blas; I. LlinaresJ. Alom, Oxidative stress in Alzheimer's disease and mild cognitive impairment with high sensitivity and specificity. *J Alzheimers Dis,* **2013**. 33,823-829.
19. Rembach, A.; J.D. Doecke; B.R. Roberts; A.D. Watt; N.G. Faux; I. Volitakis; K.K. Pertile; R.L. Rumble; B.O. Trounson; C.J. Fowler; W. Wilson; K.A. Ellis; R.N. Martins; C.C. Rowe; V.L. Villemagne; D. Ames; C.L. Masters; A.r. groupA.I. Bush, Longitudinal analysis of serum copper and ceruloplasmin in Alzheimer's disease. *J Alzheimers Dis,* **2013**. 34,171-182.
20. Alsadany, M.A.; H.H. Shehata; M.I. MohamadR.G. Mahfouz, Histone deacetylases enzyme, copper, and IL-8 levels in patients with Alzheimer's disease. *Am J Alzheimers Dis Other Demen,* **2013**. 28,54-61.
21. Brewer, G.J.; S.H. Kanzer; E.A. Zimmerman; D.F. Celmins; S.M. HeckmanR. Dick, Copper and ceruloplasmin abnormalities in Alzheimer's disease. *Am J Alzheimers Dis Other Demen,* **2010**. 25,490-497.
22. Baum, L.; I.H. Chan; S.K. Cheung; W.B. Goggins; V. Mok; L. Lam; V. Leung; E. Hui; C. Ng; J. Woo; H.F. Chiu; B.C. Zee; W. Cheng; M.H. Chan; S. Szeto; V. Lui; J. Tsoh; A.I. Bush; C.W. LamT. Kwok, Serum zinc is decreased in Alzheimer's disease and serum arsenic correlates positively with cognitive ability. *Biometals,* **2010**. 23,173-179
23. Arnal, N.; D.O. Cristalli; M.J. de AlanizC.A. Marra, Clinical utility of copper, ceruloplasmin, and metallothionein plasma determinations in human neurodegenerative patients and their first-degree relatives. *Brain Res,* **2010**. 1319,118-130.
24. Vural, H.; H. Demirin; Y. Kara; I. ErenN. Delibas, Alterations of plasma magnesium, copper, zinc, iron and selenium concentrations and some related erythrocyte antioxidant enzyme activities in patients with Alzheimer's disease. *J Trace Elem Med Biol,* **2010**. 24,169-173.
25. Agarwal, R.; S.S. Kushwaha; C.B. Tripathi; N. SinghN. Chhillar, Serum copper in Alzheimer’s disease and vascular dementia *Indian J Clin Bioch,* **2008**. 23 369-374.
26. Zappasodi, F.; C. Salustri; C. Babiloni; E. Cassetta; C. Del Percio; M. Ercolani; P.M. RossiniR. Squitti, An observational study on the influence of the APOE-epsilon4 allele on the correlation between 'free' copper toxicosis and EEG activity in Alzheimer disease. *Brain Res,* **2008**. 1215,183-189.
27. Gerhardsson, L.; T. Lundh; L. MinthonE. Londos, Metal concentrations in plasma and cerebrospinal fluid in patients with Alzheimer's disease. *Dement Geriatr Cogn Disord,* **2008**. 25,508-515.
28. Sevym S, U.O., Tamer I, Doğu O, Ozge A., Can serum levels of copper and zinc distinguish Alzheimer’s patients from normal subjects? *Journal of Neurological Sciences (Turkish),* **2007**. 24,197-205.
29. Sedighi, B.; M.A. ShafaM. Shariati, A study of serum copper and ceruloplasmin in Alzheimer’s disease in Kerman, Iran. *Neurology Asia,* **2006**. 11,107-109.
30. Bocca, B.; G. Forte; F. Petrucci; A. Pino; F. Marchione; G. Bomboi; O. Senofonte; F. GiubileiA. Alimonti, Monitoring of chemical elements and oxidative damage in patients affected by Alzheimer's disease. *Ann Ist Super Sanita,* **2005**. 41,197-203.
31. Smorgon, C.; E. Mari; A.R. Atti; E. Dalla Nora; P.F. Zamboni; F. Calzoni; A. PassaroR. Fellin, Trace elements and cognitive impairment: an elderly cohort study. *Arch Gerontol Geriatr Suppl,* **2004**.393-402.

32. Ozcankaya, R.N. Delibas, Malondialdehyde, superoxide dismutase, melatonin, iron, copper, and zinc blood concentrations in patients with Alzheimer disease: cross-sectional study. *Croat Med J,* **2002**. 43,28-32.
33. Squitti, R.; D. Lupoi; P. Pasqualetti; G. Dal Forno; F. Vernieri; P. Chiovenda; L. Rossi; M. Cortesi; E. CassettaP.M. Rossini, Elevation of serum copper levels in Alzheimer's disease. *Neurology,* **2002**. 59,1153-1161.
34. Gonzalez, C.; T. Martin; J. Cacho; M.T. Brenas; T. Arroyo; B. Garcia-Berrocal; J.A. NavajoJ.M. Gonzalez-Buitrago, Serum zinc, copper, insulin and lipids in Alzheimer's disease epsilon 4 apolipoprotein E allele carriers. *Eur J Clin Invest,* **1999**. 29,637-642.
35. Molina, J.A.; F.J. Jimenez-Jimenez; M.V. Aguilar; I. Meseguer; C.J. Mateos-Vega; M.J. Gonzalez-Munoz; F. de Bustos; J. Porta; M. Orti-Pareja; M. Zurdo; E. BarriosM.C. Martinez-Para, Cerebrospinal fluid levels of transition metals in patients with Alzheimer's disease. *J Neural Transm,* **1998**. 105,479-488.
36. Snaedal, J.; J. Kristinsson; S. Gunnarsdottir; Olafsdottir; M. BaldvinssonT. Johannesson, Copper, ceruloplasmin and superoxide dismutase in patients with Alzheimer's disease . a case-control study. *Dement Geriatr Cogn Disord,* **1998**. 9,239-242.
37. Molaschi, M.; M. Ponzetto; B. Bertacna; E. BerrinoE. Ferrario, Determination of selected trace elements in patients affected by dementia. *Arch Gerontol Geriatr,* **1996**. 22 Suppl 1,39-42.
38. Mattiello, G.; M. Gerotto; M. Favarato; F. Lazzri; G. Gasparoni; L. Gomirato; G. Mazzolini; G. Scarpa; V. Zanaboni; M.G. PiloneP.F. Zatta, Plasma Microelement Analysis from Alzheimer's and Multi-infartual Dementia Patients *Alzheimer's Diseases: Advances in Clinical and Basic Research,* **1993**.
39. Basun, H.; L.G. Forssell; L. WetterbergB. Winblad, Metals and trace elements in plasma and cerebrospinal fluid in normal aging and Alzheimer's disease. *J Neural Transm Park Dis Dement Sect,* **1991**. 3,231-58.
40. Jeandel, C.; M.B. Nicolas; F. Dubois; F. Nabet-Belleville; F. PeninG. Cuny, Lipid peroxidation and free radical scavengers in Alzheimer's disease. *Gerontology,* **1989**. 35,275-82.
41. Kapaki, E.; J. SegditsaC. Papageorgiou, Zinc, copper and magnesium concentration in serum and CSF of patients with neurological disorders. *Acta Neurol Scand,* **1989**. 79,373-378..
42. Shore, D.; R.I. Henkin; N.R. Nelson; R.P. AgarwalR.J. Wyatt, Hair and serum copper, zinc, calcium, and magnesium concentrations in Alzheimer-type dementia. *J Am Geriatr Soc* **1984**. 32,892-895.
